# Early Bactericidal Activity of Delpazolid (LCB01-0371) in Patients with Pulmonary Tuberculosis

**DOI:** 10.1128/aac.01684-21

**Published:** 2022-02-15

**Authors:** Ju Sang Kim, Yong-hyun Kim, Sang Haak Lee, Yee Hyung Kim, Jin-woo Kim, Ji Young Kang, Sung Kyoung Kim, Seung Joon Kim, Yun-Seong Kang, Tae-hyung Kim, Jeongha Mok, Min Kwang Byun, Hye Jung Park, Joon-sung Joh, Yong Bum Park, Hyeong-Seok Lim, Hongjo Choi, Seung Heon Lee, Hyejin Kim, Jeongseong Yang, Hyunji Kim, Xianlin Shen, Abdullah Alsultan, InSook Cho, Lawrence Geiter, Tae Sun Shim

**Affiliations:** a Division of Pulmonary and Critical Care Medicine, Department of Internal Medicine, Incheon St. Mary’s Hospital, College of Medicine, The Catholic University of Korea, Seoul, South Korea; b Division of Pulmonary, Allergy and Critical Care Medicine, Department of Internal Medicine, Bucheon St. Mary’s Hospital, College of Medicine, The Catholic University of Korea, Seoul, South Korea; c Division of Pulmonary, Critical Care and Sleep Medicine, Department of Internal Medicine, Eunpyeong St. Mary’s Hospital, College of Medicine, The Catholic University of Korea, Seoul, South Korea; d Department of Pulmonary, Allergy and Critical Care Medicine, Kyung Hee University Hospital at Gangdong, Kyung Hee University, Seoul, South Korea; e Division of Pulmonology and Critical Care Medicine, Department of Internal Medicine, Uijeongbu St. Mary’s Hospital, College of Medicine, The Catholic University of Korea, Seoul, South Korea; f Division of Pulmonary, Allergy and Critical Care Medicine, Department of Internal Medicine, Seoul St. Mary’s Hospital, College of Medicine, The Catholic University of Korea, Seoul, South Korea; g Division of Pulmonology, Department of Internal Medicine, St. Vincent's Hospital, College of Medicine, The Catholic University of Korea, Suwon, South Korea; h Division of Pulmonology and Critical Care Medicine, Dongguk University Ilsan Hospital, Dongguk University College of Medicine, Goyang, South Korea; i Department of Internal Medicine, Hanyang University College of Medicine, Guri, South Korea; j Department of Internal Medicine, Pusan National University Hospital, Busan, South Korea; k Department of Internal Medicine, Gangnam Severance Hospital, Yonsei University College of Medicine, Gangnam-gu, Seoul, South Korea; l Department of Internal Medicine, National Medical Center, Seoul, South Korea; m Department of Internal Medicine, Hallym University Medical Center, Kangdong Sacred Heart Hospital, Seoul, South Korea; n Department of Clinical Pharmacology and Therapeutics, Asan Medical Center, University of Ulsan College of Medicine, Seoul, South Korea; o Department of Preventive Medicine, Konyang University College of Medicine, Daejon, South Korea; p The Korean Institute of Tuberculosis, Cheongju, South Korea; q Merlin Clinical Service, Gaithersburg, Maryland, USA; r Clinical Pharmacy, Department College of Pharmacy, King Saud University, Riyadh, Saudi Arabia; s LegoChem BioSciences, Seoul, South Korea; t Consultant, Sunny Isles Beach, Florida, USA; u Department of Pulmonary and Critical Care Medicine, Asan Medical Center, University of Ulsan College of Medicine, Seoul, South Korea

**Keywords:** early bactericidal activity, *Mycobacterium tuberculosis*, experimental therapeutics, oxazolidinones

## Abstract

Delpazolid, an oxazolidinone, has been studied in non-clinical studies of efficacy and toxicity and Phase 1 clinical studies. Delpazolid has *in vitro* activity against Gram-positive bacteria, including Mycobacterium tuberculosis. This study evaluated the bactericidal activity, safety, and pharmacokinetics of delpazolid in patients with pulmonary tuberculosis (TB). Seventy-nine subjects, aged 19 to 75 years with newly diagnosed smear-positive TB with no prior treatment for the current episode and no confirmed resistance to rifampin or isoniazid, were randomized to receive delpazolid 800 mg once a day (QD), 400 mg twice a day (BID), 800 mg BID or 1,200 mg QD or an active control of isoniazid, rifampin, pyrazinamide, and ethambutol (HRZE) or linezolid 600 mg BID. The primary endpoint was the average daily reduction in log transformed bacterial load, assessed on 7H11 solid-media culture, from days 0 to 14. The average daily decline in log-CFU was 0.044 ± 0.016, 0.053 ± 0.017, 0.043 ± 0.016, and 0.019 ± 0.017, for the delpazolid 800 mg QD, 400 mg BID, 800 mg BID, and the 1,200 mg QD groups, respectively. The average daily decline in log-CFU was 0.192 ± 0.028 for the HRZE group and 0.154 ± 0.023 for the linezolid 600 mg BID group. Three serious adverse events (SAE) were reported, one each in the delpazolid 400 mg BID group (death due to worsening of TB at day 2), the HRZE group (hospitalization due to pleural effusion) and the linezolid group (hyperkalemia); none of the SAEs were assessed as related to study drugs. This study has been registered at ClinicalTrials.gov with registration number NCT02836483.

## INTRODUCTION

Treatment of multi-drug-resistant tuberculosis (MDR-TB: bacilli resistant to both rifampin and isoniazid) and extensively drug-resistant TB (XDR-TB: MDR-TB with additional resistance to quinolones and injectable drugs) is long, ranging from 9 to 20 months, and complicated with limited efficacy of 56% and 39% treatment success for MDR-TB and XDR-TB, respectively, reported by the WHO in 2019 (1). Treatment for MDR/XDR-TB have very high rates of adverse events, with nearly half of patients experiencing severe adverse events in a recent clinical trial (2).

The current standard 6-month regimen of isoniazid, rifampin, pyrazinamide, and ethambutol (HRZE) for drug-sensitive (DS)-TB has an efficacy of 95% under clinical trial conditions (3).

However, there are known safety issues with each of the component drugs in the standard regimen, with 9% of patients experiencing serious side effects in an observational study (4), 25% patients in the HRZE control arm experiencing grade 3 or 4 treatment-emergent adverse events and 12% discontinuing therapy due to treatment-emergent adverse events in the Phase 2B STAND trial (5) and with 7.9% discontinuing the standard regimen and 0.8% of patients meeting Hy’s law criteria in a recent study of novel 4-month regimens (6).

The introduction of bedaquiline (Bdq) in 2012 and two nitroimidazoles (delamanid [Dlm] in 2014 and pretomanid [Pa] in 2019), other new drugs in the development pipeline and existing drugs repurposed for TB treatment, especially linezolid (LZD), provide the possibility of new regimens for the treatment of both MDR- and DS-TB that will be safer, shorter, and potentially more effective.

Oxazolidinones may be important to shorten therapy without increasing the risk of reactivation. Mouse models of TB treatment have shown that regimens of Bdq, Pa, and LZD or sutezolid as short as 3 months can result in sterilization of lung tissue without relapse 3 months after the completion of treatment (7). The Nix-TB trial, an open-label, single-group study involving patients with XDR-TB and patients with MDR- TB unresponsive to treatment showed that a 6-month regimen of Bdq, Pa, and LZD resulted in 90% favorable outcome at 6 months after the end of treatment in 109 patients, supporting an approval of Pa (combined with Bdq and LZD) by the United States Food and Drug Administration (8).

Delpazolid (LCB01-0371) is an oxazolidinone under development by LegoChem Biosciences (Daejeon, South Korea) for the treatment of TB that has been shown to be active against Myobacterium tuberculosis in both *in vitro* and non-clinical *in vivo* models (9). A limitation of the use of oxazolidinones is the occurrence of mitochondrial toxicity resulting in myelosuppression and peripheral neuropathy (10). However, delpazolid has been shown in a rat model to cause less mitochondrial toxicity (11). In addition, non-clinical studies have shown that delpazolid is neither an inducer nor a substrate of a variety of the 107 CYP P450 enzymes or common transporters (12). Clinical studies including a single-ascending dose and multiple ascending dose studies have shown a good safety and toxicity profile with a dose proportional PK profile with oral formulations (12, 13). A food effect study showed no significant differences in the PK profile in fed or fasted patients (14).

Conducted after successful completion of clinical studies in healthy patients, this phase 2, 14-day early bactericidal activity (EBA) trial in newly diagnosed patients with uncomplicated pulmonary TB assessed EBA and toxicity.

## RESULTS

### Study population.

From December 2016 to July 2019, study subjects were screened at 15 hospitals in the Republic of Korea and 79 subjects, aged 23 to 74 years, with newly diagnosed smear-positive pulmonary TB, who had received no treatment for the current episode and had no confirmed resistance to rifampin or isoniazid were enrolled. The number of subjects treated at each hospital/site and their disposition in the study is shown by regimen allocation in Table 1.

**TABLE 1:** Demographics and baseline characteristics—all randomized subjects

	Delpazolid 800 mg QD (*N* = 15) *n* (%)	Delpazolid 400 mg BID (*N* = 16) *n* (%)	Delpazolid 800 mg BID (*N* = 16) *n* (%)	Delpazolid 1,200 mg QD (*N *= 16) *n* (%)	HRZE (*N *= 8) *n* (%)	LZD 600 mg BID (*N* = 8) *n* (%)
Age (yrs)						
*n*	15	16	16	16	8	8
Mean (SD)	52.0 (6.2)	52.0 (12.2)	54.0 (11.3)	51.0 (10.1)	48.0 (15.6)	53.0 (6.8)
Median	52.0	52.0	53.5	53.5	44.5	51.5
Q1, Q3	47.0, 58.0	47.0, 59.0	46.5, 63.5	48.5, 56.0	34.5, 59.0	47.0, 57.0
Min, Max	41.0, 63.0	23.0, 73.0	34.0, 72.0	26.0, 64.0	33.0, 74.0	45.0, 65.0
Gender						
Male	11 (73.3)	13 (81.3)	13 (81.3)	14 (87.5)	7 (87.5)	8 (100.0)
Female	4 (26.7)	3 (18.8)	3 (18.8)	2 (12.5)	1 (12.5)	NA
Race						
Asian	15 (100.0)	16 (100.0)	16 (100.0)	16 (100.0)	8 (100.0)	8 (100.0)
Ethnicity						
Non-Hispanic or Latino	15 (100.0)	16 (100.0)	16 (100.0)	16 (100.0)	8 (100.0)	8 (100.0)
Country						
Korean	15 (100.0)	16 (100.0)	16 (100.0)	16 (100.0)	8 (100.0)	8 (100.0)
Cavitation						
No	4 (26.7)	8 (50.0)	5 (31.3)	4 (25.0)	3 (37.5)	1 (12.5)
Yes	11 (73.3)	8 (50.0)	11 (68.8)	12 (75.0)	5 (62.5)	7 (87.5)
Height (cm)						
*n*	15	16	16	16	8	8
Mean (SD)	166.0 (8.9)	168.0 (9.8)	167.0 (10.0)	168.0 (6.8)	172.0 (7.8)	170.0 (4.9)
Median	170.0	164.5	166.2	166.9	171.4	170.3
Q1, Q3	159.0, 172.7	160.9, 177.9	158.7, 176.8	162.1, 172.9	166.0, 177.0	166.0, 174.2
Min, Max	153.0, 181.0	156.0, 184.0	149.0, 180.0	158.0, 180.0	162.0, 185.0	164.0, 177.0
Weight (kg)						
*n*	15	6	16	16	8	8
Mean (SD)	57.0 (7.6)	62.0 (15.1)	59.0 (12.6)	61.0 (12.1)	65.0 (14.6)	57.0 (9.5)
Median	59.0	58.1	58.0	61.5	69.5	54.0
Q1, Q3	50.1, 64.0	50.9, 76.8	50.7, 65.0	49.8, 69.1	52.0, 75.8	51.5, 61.8
Min, Max	46.0, 70.0	40.0, 89.0	34.0, 85.0	43.0, 85.0	42.0, 81.0	48.0, 77.0
Body mass index (kg/m²)						
*n*	15	16	16	16	8	8
Mean (SD)	21.0 (2.4)	22.0 (3.4)	21.0 (3.6)	22.0 (3.6)	22.0 (4.7)	20.0 (2.5)
Median	21.1	22.1	21.2	21.3	22.8	19.6
Q1, Q3	19.7, 22.6	18.3, 24.5	19.1, 22.3	18.7, 24.0	19.1, 25.2	18.4, 20.7
Min, Max	16.0, 24.0	17.0, 27.0	15.0, 29.0	17.0, 27.0	13.0, 28.0	16.0, 25.0

**FIG 1:**
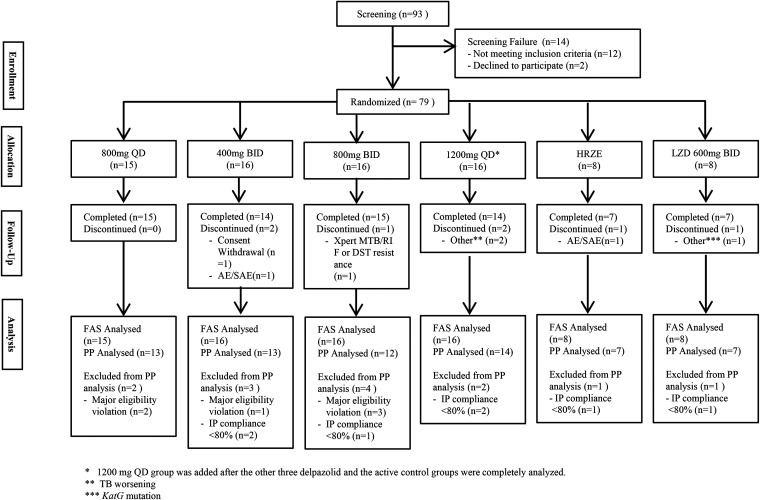
Disposition of study subjects screened and randomized.

As shown in Fig. 1 and 15, subjects were randomized to receive delpazolid 800 mg QD for 14 days, 16 patients each were randomized to receive delpazolid 400 mg BID, 800 mg BID, or 1,200 mg BID and eight patients each were randomized to receive HRZE or LZD 600 mg BID. The baseline and demographic characteristics of the randomized patients were similar in the six randomization groups and are shown in Table 2. Seven randomized subjects discontinued the study before completion with the numbers and reasons for discontinuation are shown in Fig. 1. All 79 randomized subjects were included in the full analysis set (FAS) for the intention-to-treat analysis. All 79 subjects received at least one dose of investigational medicinal product (IMP) and were included in the safety analysis set (SS). Sixty-six subjects were included in the per protocol (PP) analysis population with the number and reason for exclusion from the PP population by regimen shown in Fig. 1.

**TABLE 2:** Regimen allocation by study hospital/site

Hospital/site name	Regimen allocation						
	800 QD	400 BID	800 BID	1,200 QD	HRZE	LZD	Total
The Catholic University of Korea Seoul St. Mary's Hospital	2	3	4	1	0	1	11
Gang Nam Severance Hospital	0	0	3	0	0	0	3
Gangdong Kyunghee University Hospital	2	2	0	1	1	1	7
National Medical Center	0	0	0	0	0	1	1
Dongguk University Ilsan Hospital	0	1	0	4	0	0	5
Pusan National University Hospital	0	0	0	1	1	1	3
Bucheon St. Mary's Hospital	4	2	2	2	0	0	10
Seoul Asan Hospital	0	0	0	1	1	0	2
St. Paul's Hospital	0	3	2	1	1	1	8
St. Vincent's Hospital	1	0	0	5	0	0	6
Ulsan University Hospital	1	0	1	0	0	0	2
Uijeongbu St. Mary's Hospital	0	3	1	0	2	1	7
Incheon St. Mary's Hospital	4	1	2	0	2	2	11
Hanyang University Guri Hospital	1	1	1	0	0	0	3
Grand total	15	16	16	16	8	8	79

Patients could be treated either as inpatients or outpatients with directly observed therapy (DOT) for the outpatients. At all but two of the sites the proportion of patients treated as inpatients was at least 67% and at seven of the 14 sites 100% of patients managed as inpatients. At one site, 100% of patients were managed as outpatients and at another 18% were managed as inpatients. By regimen, 40% of patients randomized to the delpazolid 800 mg QD regimen were managed as inpatients and the proportion managed as inpatients for the other regimens was very similar, ranging from 67% to 75%. Table S3 in the supplemental materials shows the distribution by site, regimen and management.

### Bactericidal activity.

The primary efficacy endpoint was the EBA over 0 to 14 days, defined as the average daily reduction in log transformed bacterial load, assessed on solid media cultures from day 0 to day 14. Treatment difference on the average daily reduction were assessed using a mixed model repeated measure (MMRM) statistical method with an unstructured covariance structure. The EBA over the periods 0 to 2 days, 2 to 14 days, and 2 to 7 days was also assessed as secondary endpoints.

**FIG 2:**
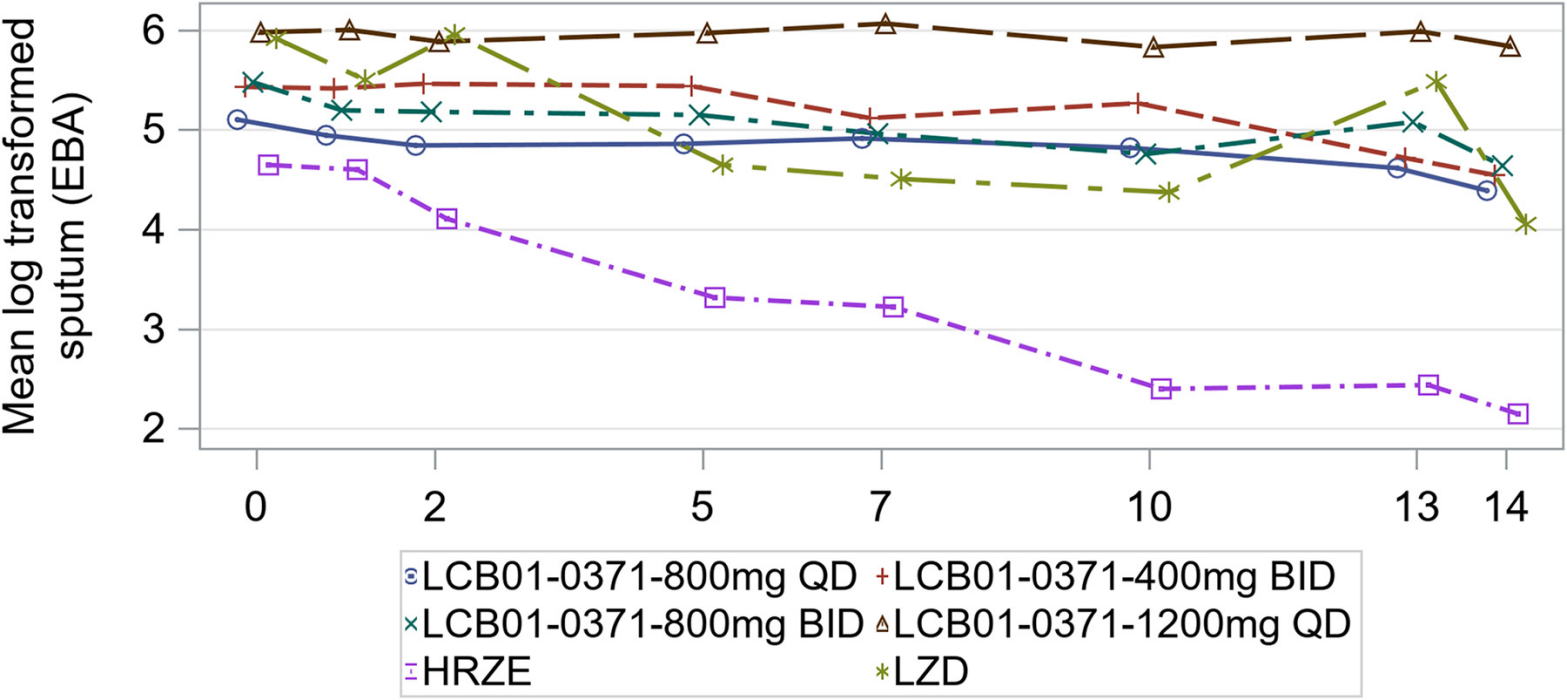
Results for each regimen group.

The EBA results in the FAS by treatment group are shown in Table 3 and the results for each regimen group are graphed in Fig. 2 for the 14-day time period. For the primary endpoint, EBA 0 to 14, the daily change in log-CFU (log-CFU per day was −0.044, −0.053, −0.043, −0.019, −0.192, and 0.−154 for the delpazolid 800 mg QD, 400 mg BID, 800 mg BID, 1,200 mg QD, HRZE and LZD 600 mg groups, respectively). The decline in log-CFU as a percentage of the HRZE group was 22.9%, 27.6%, 22.4%, and 9.9% for the delpazolid 800 mg QD, 400 mg BID, 800 mg BID, and 1,200 mg QD groups, respectively. The decline in log-CFU in the LZD 600 mg BID group was 0.154, 80.2% of the decline in the HRZE group. The decline in log-CFU and the decline as a percentage of the HRZE response was nearly identical for the PP group. A graph of the log transformed mean daily decline for each regimen and a table of the EBA 0 to 14 for each regimen is shown in the supplementary data.

**TABLE 3:** Summary of daily log transformed sputum (EBA) fall (slope) in time intervals for patients in the full analysis set (FAS)

Days	Statistics	LCB01-0371				Control group	
		800 mg QD (*N* = 15) Est. (S.E)^2^	400 mg BID (*N* = 16) Est. (S.E)^2^	800 mg BID (*N* = 16) Est. (S.E)^2^	1200mg QD (*N* = 16) Est. (S.E)^2^	HRZE (*N* = 8) Est. (S.E)^2^	Zyvox 600 mg (*N* = 8) Est. (S.E)^2^
0 to 2	Daily fall	–0.132 (0.113)	0.016 (0.113)	–0.145 (0.110)	–0.047 (0.113)	–0.273 (0.155)	0.023 (0.155)

2 to 7	Daily fall	0.012 (0.035)	–0.077 (0.035)	–0.054 (0.035)	0.040 (0.035)	–0.220 (0.051)	–0.213 (0.055)

2 to 14	Daily fall	–0.041 (0.018)	–0.067 (0.019)	–0.049 (0.018)	–0.017 (0.020)	–0.170 (0.031)	–0.143 (0.028)

0 to 14	Daily fall	–0.044 (0.016)	–0.053 (0.017)	–0.043 (0.016)	–0.019 (0.017)	–0.192 (0.028)	–0.154 (0.023)

Change in time-to-detection (TTD) in the MGIT system was another measure of decline in bacterial load and is shown in Table 4; an increase in TTD indicates a decline in bacterial load. The daily change in TTD (hours) for the period 0 to 14 days for the FAS was 2.7, 0.3, 2.3, 0.7, and 2.9 for the delpazolid 800 mg QD, 400 mg BID, 800 mg BID, 1,200 mg QD groups, and the linezolid 600 mg BID group, respectively. The change in TTD as a percentage of the HRZE group was 17.4%, 1.9%, 15.1%, 4.4%, and 18.6% for the delpazolid 800 mg QD, 400 mg BID, 800 mg BID, 1,200 mg QD groups, and the LZD 600 mg BID group, respectively. The daily change in TTD (hours) or the period 0 to 14 days for the PP set was 1. 4, 1.8, 2.0, 0.1, and 6.5 and 9.8%, 11.9%, 13.8%, 0.7%, and 44.3% for the delpazolid 800 mg QD, 400 mg BID, 800 mg BID, 1,200 mg QD groups, and the LZD 600 mg BID group, respectively.

**TABLE 4:** Summary of mean daily change in time (days) to detection in the MGIT system in defined intervals for patients in the full analysis set (FAS)

Days	Statistics^3^	LCB01-0371				Control group	
		800 mg QD (*N* = 15) Est. (S.E)^2^	400 mg BID (*N* = 16) Est. (S.E)^2^	800 mg BID (*N* = 16) Est. (S.E)2	1,200 mg QD (*N* = 16) Est. (S.E)^2^	HRZE (*N* = 8) Est. (S.E)^2^	LZD 600 mg BID (*N* = 8) Est. (S.E)^2^
0 to 2	Mean daily change	3.60 (15.89)	–29.85 (15.38)	0.94 (15.38)	–0.41 (15.38)	5.81 (21.76)	56.06 (21.76)

2 to 7	Mean daily change	–1.38 (3.94)	1.71 (4.03)	2.94 (3.89)	0.04 (3.89)	1.31 (5.39)	0.33 (5.62)

2 to 14	Mean daily change	3.04 (2.47)	1.87 (2.53)	3.08 (2.42)	0.94 (2.48)	14.97 (3.34)	3.34 (3.55)

0 to 14	Mean daily change	2.69 (2.32)	0.30 (2.32)	2.33 (2.25)	0.68 (2.29)	15.42 (3.13)	2.87 (3.31)

### Safety.

All 79 randomized subjects took at least one dose of study medication and were included in the safety set for analysis. The mean duration of IMP exposure was similar in the six treatment groups and ranged from 12.5 to 14.0 days.

Adverse events (AE) are summarized in Table 5. The proportion of patients experiencing an AE was 26.7%, 75.0%, 43.8%, and 62.5% in the delpazolid 800 mg QD, 400 mg BID, 800 mg BID, and the 1,200 mg QD groups, respectively. In the HRZE and LZD 600 mg BID, the proportion of subjects experiencing an AE was 87.5% and 62.5%, respectively.

**Table 5:** Summary of adverse events—safety set

Category	800 mg QD (*N* = 15)		400 mg BID (*N* = 16)		800 mg BID (*N* = 16)		1,200 mg QD (*N* = 16)		HRZE (*N* = 8)		LZD 600 mg BID (*N* = 8)	
	*n* (%)	[count]	*n* (%)	[count]	*n* (%)	[count]	*n* (%)	[count]	*n* (%)	[count]	*n* (%)	[count]
Treatment-emergent adverse event (TEAE)	4 (26.67)	[12]	12 (75.00)	[23]	7 (43.75)	[13]	10 (62.50)	[22]	7 (87.50)	[19]	5 (62.50)	[16]
Adverse drug reaction (ADR)	0	0	3 (18.75)	[8]	3 (18.75)	[6]	6 (37.50)	[13]	4 (50.00)	[9]	4 (50.00)	[7]
Serious adverse event (SAE)	0	0	1 (6.25)	[1]	0	0	0	0	1 (12.50)	[1]	1 (12.50)	[1]
Death	0	0	1 (100.00)	[1]	0	0	0	0	0	0	0	0
Life threatening	0	0	0	0	0	0	0	0	0	0	0	0
Hospitalization or prolongation of existing hospitalization	0	0	0	0	0	0	0	0	1 (100.00)	[1]	0	0
Persistent or significant disability/incapacity	0	0	0	0	0	0	0	0	0	0	0	0
Congenital anomaly or birth defect	0	0	0	0	0	0	0	0	0	0	0	0
Other medically serious adverse events	0	0	0	0	0	0	0	0	0	0	1 (100.00)	[1]
Serious adverse drug reaction (SADR)	0	0	0	0	0	0	0	0	0	0	0	0
Death	0	0	0	0	0	0	0	0	0	0	0	0
Life threatening	0	0	0	0	0	0	0	0	0	0	0	0
Hospitalization or prolongation of existing hospitalization	0	0	0	0	0	0	0	0	0	0	0	0
Persistent or significant disability/incapacity	0	0	0	0	0	0	0	0	0	0	0	0
Congenital anomaly or birth defect	0	0	0	0	0	0	0	0	0	0	0	0
Other medically serious adverse events	0	0	0	0	0	0	0	0	0	0	0	0

The most frequently reported AEs were gastrointestinal disorders, especially nausea, diarrhea, and vomiting.

The proportion of study subjects with adverse drug reactions (ADR’s), which are likely or probably related to the study drug, was 0%, 18.8%, 18.8%, and 37.5% in the delpazolid 800 mg QD, 400 mg BID, 800 mg BID, and the 1,200 mg QD groups, respectively. The proportion of patients experiencing an ADR was 50% in both the HRZE and LZD 600 mg BID groups.

The most frequently reported ADRs were gastrointestinal disorders, especially nausea and diarrhea.

There were four subjects with treatment emergent adverse events (TEAE’s) leading to withdrawal; one subject each in the delpazolid 400 mg BID and 1,200 mg QD groups experienced worsening TB that was considered unlikely related to the study drug and one subject in the HRZE was withdrawn due to increased alanine aminotransferase (ALT) and aspartate aminotransferase (AST) that was assessed as “probably related to the study drug.”

The severity of most of AEs and ADRs were mild, and there were no grade 4 or 5 ADRs.

One subject randomized to the delpazolid 400 mg BID group died during the study, but the death was judged not related to the study drug, although an autopsy was not performed. The subject was a 53-year-old male with a BMI of 16.5 kg/m^2^. The TB disease severely worsened after 1 day and the delpazolid was discontinued on day 2 before the second dose of delpazolid was administered. The patient was then started on an HRZE regimen but the disease continued to worsen and the patient died on study-day 6.

## DISCUSSION

Oxazolidinones are likely to be an important part of the development of new regimens for TB treatment that are short and well tolerated. The new drug pretomanid has been approved for use in a 6- to 9-month all-oral regimen for XDR- and treatment resistant MDR-TB when administered with Bdq and an oxazolidinone, LZD (8). The major limitation of LZD, is the association with mitochondrial toxicity (10); oxazolidinones have also been associated with serotonin syndrome (15).

This study has shown that, in an EBA trial, monotherapy with delpazolid is efficacious against M. tuberculosis, achieving about 25% of the reduction in log-CFU in sputum, compared with reduction in the group given the four-drug HRZE regimen, when given at a dose of 800 mg QD, 400 mg BID, or 800 mg BID. This is somewhat less than has been observed with LZD given at a dose of 600 mg or higher but greater than that achieved with a dose of 300 mg QD and 300 mg BID (16). LZD at these doses has been shown to provide clinically significant benefit in the treatment of MDR-TB while also resulting in significant toxicity, making the confirmation of lower toxicity with delpazolid an important point to investigate.

While this study shows bactericidal efficacy against M. tuberculosis, it is important to note that delpazolid has shown the potential to be much less toxic than other oxazolidinones. A hollow-fiber model study by Brown et al. (17) showed that mitochondrial toxicity is associated with lower drug trough levels. Delpazolid half-life is one third to one half of the LZD half-life and delpazolid is undetectable within 12 h, at the doses studied in this trial.

Phase 1 and non-clinical studies show that delpazolid causes less mitochondrial toxicity and neither substrate or inducer of transporters or CYP enzymes compared with LZD and sutezolid. This decreases the likelihood of drug-drug interactions and decreases the likelihood of the serotonin syndrome observed with other oxazolidinones (15).

There are several potential limitations of this study. While most EBA studies are conducted at a small number of centers, this trial was conducted at 14 medical centers, making standardization very difficult. Further complicating standardization, six of the sites enrolled only one to four subjects. However, it is important to note that all sputum specimens were processed and cultured at the Korean Institute of Tuberculosis, Korean National Tuberculosis Association in Cheongju, South Korea. The standard deviation and coefficient of variability around the colony counts and time to detection in the MGIT system were comparable with other EBA studies that included and HRZE active control regimen (18–20).

The low EBA estimate for the 1,200 mg QD dose, relative to the other delpazolid doses has not been explained. The subjects in this dose group were enrolled at eight different treatment centers, so it is unlikely that there was a treatment center effect. Neither the presence of cavities nor baseline bacterial load correlated with EBA or enrollment in the 1,200 mg QD group.

There was no dose response curve observed as all dose groups except the 1,200 mg group had very similar estimates of EBA as assessed by changes in colony counts changes in TTD in the MGIT system. Also, the rank order of the EBA_0-14_ estimated by colony counts and changes in TTD were not consistent. For example, the 400 mg BID group had the greatest EBA assessed by changes in colony counts but the lowest EBA assessed by changes in TTD.

While the results of this trial, showing about 25% of the EBA achieved by the HRZE regimen, are encouraging, questions remain about dosing and the efficacy and safety of delpazolid in a regimen with other oral drugs. Many of these answers should come from a study sponsored by LegoChem Biosciences and implemented by the Pan-African Consortium for the Evaluation of Antituberculosis Antibiotics (PanACEA) titled DElpazolid dose-finding and Combination Development (DECODE). In this trial, patients will be randomized to receive bedaquiline, delamanid, and moxifloxacin or the same three drugs plus different doses of delpazolid for 4 months. Patients who achieve sputum culture conversion within 2 months will have therapy discontinued and then be followed for relapse. DECODE is designed to provide information on exposure-response, safety, and on the contribution of delpazolid a regimen likely to be very efficacious and well tolerated. This study is similar in design and will be conducted at the same sites as a trial of sutezolid (SUDOCU) in combination with Bdq, Dlm, and moxifloxacin. The similarity in design of DECODE and SUDOCU will allow for a direct comparison of efficacy and toxicity of delpazolid and sutezolid. In addition to the clinical trial, additional mouse modeling of delpazolid in regimens with other new drugs and hollow-fiber system modeling of exposure-response (both efficacy and mitochondrial toxicity) will guide dosing in pivotal trials.

## MATERIALS AND METHODS

### Study design and subjects.

This study was a phase 2, multicenter, randomized, open-labeled, active-controlled clinical trial (ClinicalTrials.gov identifier NCT02836483). The primary objective of the study was to investigate the anti-TB activity of delpazolid through the assessment of the extended EBA over 14 days. TB bacilli were tested for resistance to isoniazid and rifampicin with the MTBDRplus line probe assay. Subjects aged 19 to 75 years with newly diagnosed smear-positive pulmonary TB who had received no treatment for the current episode and had no confirmed resistance to rifampin or isoniazid by a katG mutations were enrolled at one of 15 hospitals in the Republic of Korea. Subjects were randomized to receive the study drug, delpazolid, at one of four doses; 800 mg QD, 400 mg BID, 800 mg BID, or 1,200 mg once daily; or to receive an active control treatment of isoniazid, rifampin, pyrazinamide, and ethambutol at standard doses or LZD 600 mg BID. Study subjects were admitted to the hospitals where they were enrolled or treated at home with directly observed therapy of study medication. Study subjects were examined for eligibility for up to 14 days and were then seen on days 1, 2, 3, 6, 8, 11, and 14 for collection of an overnight sputum specimen and safety assessments on days 1, 8, and 15. All subjects gave written informed consent, and the protocol and informed-consent form were approved by the Ministry of Food and Drug Safety and the Institutional Review Board of each participating sites.

### Mycobacteriology.

Subjects were instructed to provide a 12-h, overnight sputum collection on scheduled days to the DOT worker who gives them their TB medication. The subjects were further instructed to brush their teeth after the evening meal and then begin the 12-h collection period. The difference between sputum and saliva was described to the patients at the beginning of the study and subjects were asked to try to produce 10 mL (about 2 tablespoons) of sputum for each overnight collection period. The subjects were instructed to only use the sterile collection cups provided to them and to store collected sputum in the refrigerator until it was given to the DOT worker. The procedures were the same for hospitalized and subjects managed as outpatients except that the collection was overseen by a study nurse, instead of a DOT worker.

The DOT worker took the overnight specimens to the hospital and they were stored there until sent to the KNTA laboratory. In most cases the sputum was received by the KNTA laboratory the same day it was collected from the patient; 498 of the 695 specimens (72%) collected were shipped the same day as collected. If not shipped on that day, the sputum samples were kept in a refrigerator until shipped. Shipment and storage at the hospital, data on collection. It is important to note that the mean volume of sputum collected was 8.98 mL for both the inpatient and outpatient subjects. Further 41% of outpatients and 40% of inpatients produced specimens that were >10 mL.

Solid media cultures for CFU (CFU counts, TTD in the MGIT, and RNA detection were performed using standardized protocols). For CFU counting, sputum was homogenized with magnetic bead stirring. Sputum samples were homogenized with 0.5% Dithiothreitol. Homogenized sputum was plated on 7H11 agar medium with added Polymixin B, Amphotericin B, Carbenicillin, and Trimethorprim (PACT) antibiotics to inhibit the growth of contaminants in sputum. Undiluted, homogenized sputum and five serial 10-fold dilutions were plated, and colony counting was done up to 21 days on Ogawa medium. An undiluted, homogenized specimen was inoculated in liquid media for assessment of time to detection in a single Bactec MGIT 960 system up to 42 days. Undiluted, homogenized sputum was stored at −70°C for storage for backup analysis and later RNA extraction. All sputum processing and analysis were undertaken at the Korean Institute of Tuberculosis and detailed procedures used by the laboratory are included in the supplemental material.

### Statistical analyses.

Data analyzed were assessed in four different sets: the full analysis set (FAS-subjects, who were randomized, administered IMP at least once and analyzable for EBA endpoints; subjects were analyzed by the treatment assignment and was the primary analysis population for all EBA analyses); the per-protocol set (PPS-FAS subjects without important protocol deviation. EBA analyses were repeated in the PPS); the pharmacokinetic set (PKS-subjects, who were randomized, administered the IMP and had at least 1 drug concentration data and was used for all PK analyses, unless specified otherwise), and the safety set (subjects who were randomized and administered at least one IMP). Subjects were analyzed by the treatment actually received. The safety set was used for all safety analyses, unless specified otherwise.

### Subjects with the following important protocol deviations were excluded from the PPS.

Inclusion/exclusion criteria violation that could seriously distort the EBA or safety assessment of the IMP or affect subject safety, in the opinion of the Investigator or sponsor, were use of other pharmacological or surgical treatment for the study indication during the trial; use of prohibited concomitant medication; no washout of previously specified drugs prior to randomization or IMP compliance <80% (however, it is important to note that no patients missed more than one dose and were therefore >95% compliant).

All data measured and recorded in this study was summarized by using appropriate analysis sets defined above. For descriptive statistics, mean, standard deviation (SD), median, minimum, and maximum were presented for continuous variables, and frequency and percentage were provided for categorical variables in principle. Although this was an exploratory and descriptive study, a significance test was conducted for therapeutic effects in delpazolid groups and between delpazolid groups and control groups. For the primary efficacy endpoint, a two-sided test was conducted at a 10% significance level, and for all other statistical tests, a two-sided test was conducted at a 5% significance level.

Concomitant medications data were coded by WHO Drug Anatomical Therapeutic Chemical (ATC) 2019 class, and AE and medical history were coded by system organ class (SOC) and preferred term (PT) by using Medical Dictionary for Regulatory Activities (MedDRA) version 22.0.

### Early bactericidal activity analysis.

Summary statistics on the primary efficacy endpoint was provided for all subjects in FAS by treatment groups over time. The same summary tables were also provided for the PPS. The graphic presentations were provided by treatment groups based on the statistical analysis results over time. The tables and figures for the PPS are shown in the supplemental data.

Treatment difference on the average daily reduction in log transformed bacterial load from day 0 to day 14 was assessed using MMRM statistical method with unstructured covariance structure. No missing value was imputed on data set level since MMRM has embedded missing value imputation algorithm, assuming missing at random (MAR).

The MMRM with unequal slope model was adopted. The model included treatment groups and its interaction with assessment day (day at baseline = 0). The interaction term was used to compare the difference of the slopes among the study groups versus the controls. The cavitation on CXR, age, and sex also was included as confounding factors in the statistical model.

Study center effect was also investigated for summary statistics on the primary endpoint.

Multiple comparisons were performed between each active treatment group versus each control group as indicated in the model above. The raw *P values* from all multiple comparisons were presented without further multiplicity justifications. Based on this approach, the *P values* generated from the multiple comparisons at final analyses were for exploratory purpose; further confirmatory statistical inference might be conducted in the potential phase 3 trial, based on results from the phase 2 trial.

Additionally, for the EBA assessed with a log rate of change of the mycobacteria CFU in sputum using solid culture of tuberculosis mycobacteria EBA_0-14_ = (logCFU(day0) −logCFU(day14))/14, descriptive statistics (number of subjects, mean, SD, median, minimum, maximum) were presented for all subjects in FAS by treatment groups. Treatment effect was compared with control using a one-way analysis of variance (ANOVA) or Kruskal-Wallis test if more appropriate. Additionally, the difference between the treatment groups for the log transformed mean change in the standard EBA changed by 14 days after the administration was compared and tested using MMRM.
