# Supplementary material for: Early Bactericidal Activity of Delpazolid (LCB01-0371) in Patients with Pulmonary Tuberculosis
Source: Antimicrob Agents Chemother. 2022 Feb 15;66(2):e01684-21. doi: 10.1128/aac.01684-21 (PMC8846473; doi:10.1128/aac.01684-21)
Supplement: Supplemental file 1 — Supplemental material. Download aac.01684-21-s0001.pdf, PDF file, 0.7 MB [file aac.01684-21-s0001.pdf]

Supplemental Materials

Suplement 1. Results of Per Protocol Set (PPS) Analyses 101

1.a SF1 Mean Over Time: Log Transformed Sputum (EBA) – Per Protocol Set (Data as Observed)

1.b ST1 Summary of Daily Log Transformed Sputum (EBA) Fall (Slope) in Time – PPS

Suplement 2. ST2 Table of Distribution of Subjects by Hospital/Site by Regimen by Treatment as Inpatient vs. Outpatient 4

Suplement 3. KIT Laboratory Procedures 6

Suplement 1. Results of Per Protocol Set (PPS) Analyses

1.a SF1 Mean Over Time: Log Transformed Sputum (EBA) – Per Protocol Set (Data as

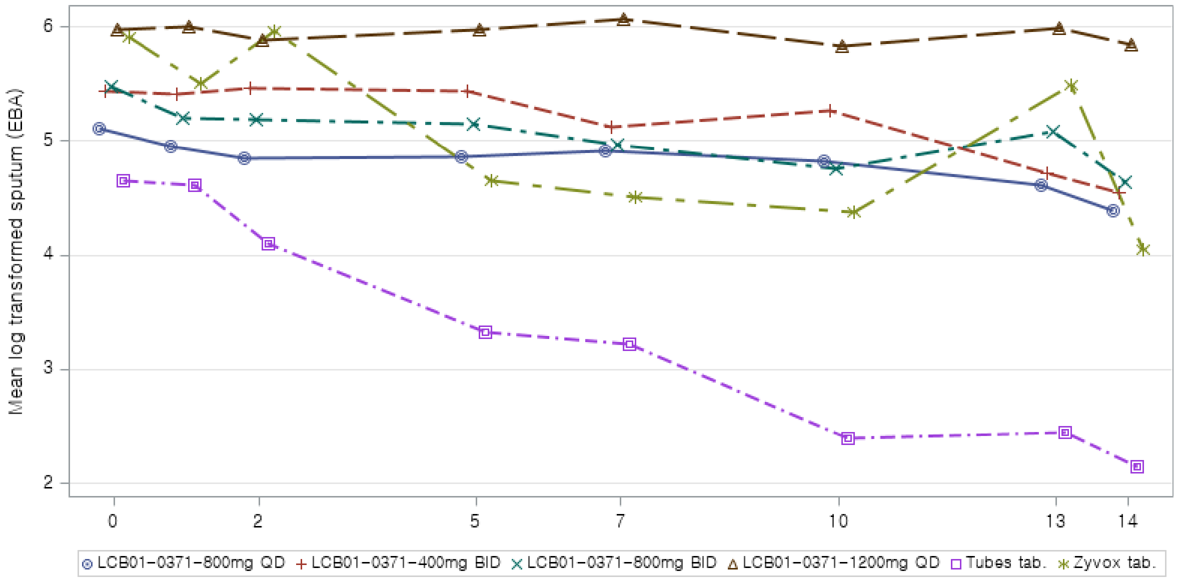

1.b ST1 Summary of Daily Log Transformed Sputum (EBA) Fall (Slope) in Time Intervals – PPS

Time period	Delpazolid 800 mg QD (N=13) Est. (S.E)	Delpazolid 400 mg BID (N=13) Est. (S.E)	Delpazolid 800 mg BID (N=12) Est. (S.E)	Delpazolid 1200 mg QD (N=14) Est. (S.E)	HRZE (N=7) Est. (S.E)	LZD 600 mg BID (N=7) Est. (S.E)
0-2 Days	-0.178 (0.119)	0.104 (0.119)	-0.244 (0.124)	-0.029 (0.119)	-0.287 (0.162)	-0.033 (0.162)
2-7 Days	0.010 (0.039)	-0.076 (0.037)	-0.031 (0.041)	0.051 (0.039)	-0.212 (0.057)	-0.202 (0.057)
2-14 Days	-0.044 (0.020)	-0.068 (0.020)	-0.033 (0.021)	-0.020 (0.020)	-0.190 (0.036)	-0.141 (0.029)
0-14 Days	-0.050 (0.017)	-0.052 (0.017)	-0.032 (0.019)	-0.019 (0.018)	-0.207 (0.032)	-0.157 (0.024)

Suplement 2. ST2 Table of Distribution of Subjects by Hospital/Site by Regimen by Treatment as Inpatient vs. Outpatient

Regimen	Delpazolid 800 mg QD			Delpazolid 400 mg BID			Delpazolid 800 mg BID			HRZE			Linezolid 600 mg QD			Delpazolid 1200mg QD			Total			Percent Inpatient
Treatment type	OUT	IN	TOT	OUT	IN	TOT	OUT	IN	TOT	OUT	IN	TOT	OUT	IN	TOT	OUT	IN	TOT	OUT	IN	TOT	
Seoul Asan Hospital	0	0	0	0	0	0	0	0	0	0	1	1	0	0	0	0	1	1	0	2	2	100%
St. Paul's Hospital	0	0	0	0	3	3	0	2	2	0	1	1	0	1	1	0	1	1	0	8	8	100%
Catholic University of Korea, Seoul St. Mary's Hospital	1	1	2	0	3	3	0	4	4	0	0	0	0	1	1	0	1	1	1	10	11	91%
Dongguk University Ilsan Hospital	0	0	0	0	1	1	0	0	0	0	0	0	0	0	0	1	3	4	1	4	5	80%
Hanyang University Guri Hospital	1	0	1	0	1	1	0	1	1	0	0	0	0	0	0	0	0		1	2	3	67%
Incheon St. Mary's Hospital	3	1	4	1	0	1	2	0	2	1	1	2	2	0	2	0	0		9	2	11	18%
Gangdong Kyunghee University Hospital	0	2	2	0	2	2	0	0	0	0	1	1	0	1	1	0	0	1	0	6	6	100%
National Medical Center	0	0	0	0	0	0	0	0	0	0	0	0	0	1	1	0	0		0	1	1	100%
Pusan National University Hospita	0	0	0	0	0	0	0	0	0	0	1	1	0	1	1	0	1	1	0	3	3	100%
St. Vincent's Hospital	0	1	1	0	0	0	0	0	0	0	0	0	0	0	0	0	5	5	0	6	6	100%
Uijeongbu St. Mary's Hospital	0	0	0	2	1	3	0	1	1	1	1	2	0	1	1	0	0		3	4	7	57%
Gang Nam Severance Hospital	0	0	0	0	0	0	1	2	3	0	0	0	0	0	0	0	0		1	2	3	67%
Ulsan University Hospital	0	1	1	0	0	0	0	1	1	0	0	0	0	0	0	0	0		0	2	2	100%
Bucheon St. Mary's Hospital	4	0	4	2	0	2	2	0	2	0	0	0	0	0	0	2	0	2	10	0	10	0%

Regimen	Delpazolid 800 mg QD			Delpazolid 400 mg BID			Delpazolid 800 mg BID			HRZE			Linezolid 600 mg QD			Delpazolid 1200mg QD			Total			Percent Inpatient
Grand Total	9	6	15	5	11	16	5	11	16	2	6	8	2	6	8	3	12	16	26	52	78	67 %
Percent Inpatient	40%			69%			69%			75%			75%			75%			67%			

IN = Inpatient treatment meaning that the patient was treated as a hospital inpatient for 15 days or more during the 28day study

OUT = Outpatient treatment meaning that the patient was treated at home with Directly Observed Therpay for 14 days or less during the 28day study

HRZE = Tuberculosis treatment with isoniazid, rifampicin,pyrazinamide and ethambutol at standard doses with a combination, fixed dose tablet Tubersol®

Suplement 3. KIT Laboratory

Evaluation of Early Bactericidal Activity (EBA) of Orally Administered LCB01-0371 in Adult Patients with AFB (Acid-Fast Bacilli) Stain-positive Pulmonary Tuberculosis

PURPOSE

To evaluate the bactericidal activity of an anti-tuberculosis drug (LCB01-0371) on *Mycobacterium tuberculosis* in lesions by a microbiological method, decreases in CFU, increases in MGIT TTD, and changes in mRNA level in sputum specimens are measured.

Specimen Flowchart

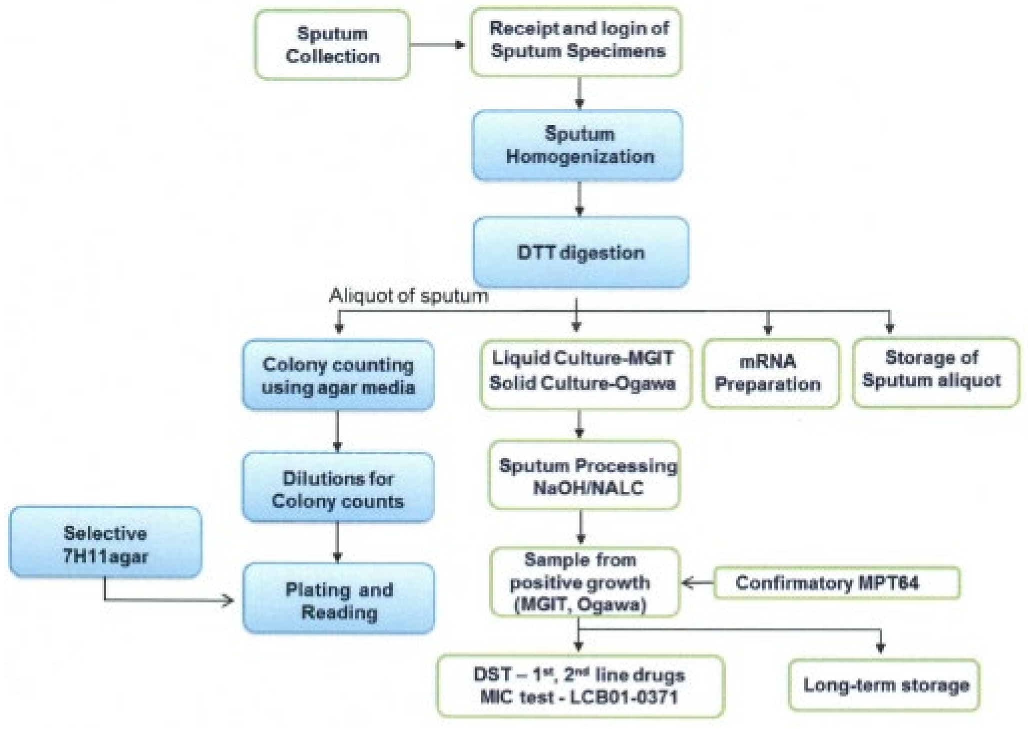

* QC procedures apply to all procedures above

I. Receipt of Sputum

1. Receipt of Sputum

① Receive triple-packed sputum specimens and a "certificate of receipt of sputum specimens" from a person in charge of transportation.

② After checking temperature and packaging states, measure internal temperature of the shipping box of sputum specimens using a non-contact infrared thermometer.

- If the internal temperature of the shipping box of sputum specimens exceeds the acceptance refrigeration temperature 2~8 °C (± 2 °C), contact the CRO representative.

③ Sign the certificate of receipt of sputum specimens. File the original in the tuberculosis research institute, and send a copy of it to Clinical Pathology Department of Yonsei University. Mail or fax a scanned copy of it the CRO representative.

④ Receive a “temperature recording paper” of the transport vehicle from the person in charge of transportation. Record sputum ID on the recording paper and file it.

⑤ Record ID of received sputum, Lab number, internal temperature of shipping box, and the date and time of receiving sputum in Specimen Processing Log.

⑥ Record the actual amount of sputum to be used in the experiment and the state of sputum, take a picture, and record the date and time of sputum homogenization in Specimen Processing Log.

2. Equipment and Documents Required

① Non-contact infrared thermometer

② Certificate of receipt of sputum specimens

③ Contact number of CRO representative

✓ Worksheets to fill out: Specimen Processing Log Form

II. Homogenization and Aliquoting of Sputum

PRINCIPLE

Although NaOH is usually used for homogenization of sputum and removal of contaminants, sputum is homogenized with Dithiothreitol (DTT) without NaOH treatment to obtain the maximum number of viable bacteria of *Mycobacterium tuberculosis*.

1. Homogenization of Sputum

① Air-dry selective 7H11 agar plates to be used in the experiment in a BSC cabinet for 1 hour according to the number of sputum specimens.

② Record the information on sputum specimens to be used in the experiment (sputum ID, lab No., etc.) and date on a labeling tape.

③ Put a magnetic spin bar of 22.2 mm x 8 mm in size into the labeled sputum container, and stir with a magnetic spin bar for 30 minutes to homogenize sputum.

- If the amount of sputum is more than 10 ml, stir for 30 minutes and transfer into a 50 ml conical tube.

④ After adding the same amount of Sputasol (0.1% DTT) to the sputum, vortex for one minute.

- The final concentration of DTT is 0.05%.

⑤ Leave the sputum to react at room temperature for 20 minutes.

2. Aliquoting of Homogenized Sputum

① In cases that the amount of sputum is insufficient, priorities are as follows:

a) Priority of experiments: CFU test > MGIT / Ogawa culture > Backup storage > RNA preparation.

② Aliquot sputum for each experiment as follows:

a) CFU test: Transfer more than 1.5 ml of homogenized sputum into a 15 ml conical tube.

b) MGIT / Ogawa culture: Transfer 5 ml of homogenized sputum to a 50 ml conical tube and proceed to the decontamination process.

c) RNA preparation: Transfer 5 ml of homogenized sputum in a 50 ml conical tube.

d) Backup storage: Transfer 1.5 ml of homogenized sputum in a cryovial and store at -70℃.

3. Reagents Required

① Preparation of Sputasol (minus DTT) 1000 ml

a) Put 7.8 g of sodium chloride, 0.2 g of potassium chloride, 1.12 g of disodium hydrogen phosphate, and 0.2 g of potassium dihydrogen phosphate in a beaker containing 800ml of D.W. After dissolving completely, add D.W. to adjust the final volume to 1000 ml.

b) After autoclaving, transfer 18 ml each into 50 ml conical tubes and store in a refrigerator (The self-life is 6 months).

② Preparation of 1.0% Dithiothreitol 100 ml

a) Dissolve 1.0 g of DTT in 100 ml of D.W. After filtration through a 0.22 um syringe filter, aliquot and store in a refrigerator (The self-life is 6 months).

③ Preparation of Sputasol (0.1% DTT)

a) Add 2 ml of 1.0% DTT into the 18 ml solution aliquoted in step ① to make the final concentration of DTT 0.1%. DTT-added Sputasol solution (0.1% DTT) should be used within 48 hours.

4. Equipment and Materials Required

① Magnetic Spin Bar of 22.2 mm x 8 mm in size

② Stirrer

③ Conical tube (15 ml, 50 ml)

④ 0.22 um syringe filter

⑤ 1.5 ml cryovial

✓ Worksheets to fill out: Refrigerator Temperature Record Form
Reagent Preparation Record Form
New Reagents/Media Form

III. Colony Counting Using Selective 7H11 Agar

Principle

Sputum homogenized with Dithiothreitol (DTT) is analyzed by EBA test using 7H11 agar medium with added Polymixin B, Amphotericin B, Carbenicillin, and Trimethorprim (PACT) antibiotics. PACT antibiotics Inhibit the growth of contaminants in sputum.

1. Dilution of Sputum

① Dilute homogenized sputum serially in Saline-Tween 80 buffer up to 10^0~10^{-5} as shown in the figure below.

- Detergent Tween 80 helps release aggregated tuberculosis bacteria in the sputum into single cells
- Open the cap with caution as a large amount of aerosol may be generated during vortexing.

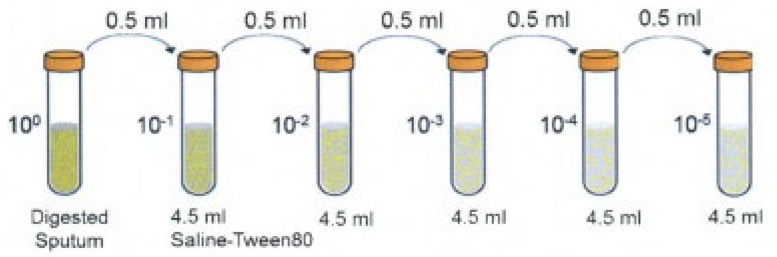

② Serial dilution procedure

a) Prepare more than 1.5 ml of homogenized sputum in a 15 ml conical tube (10^0 tube, undiluted sputum)

b) Prepare five 15 ml conical tubes, and add 4.5 ml of Saline-Tween 80 solution into each tube using a 5 ml serological pipette.

c) Using a 1 ml barrier tip, transfer 0.5 ml of sputum from the 10^0 tube to the 10^{-1} tube. Vortex for 20 seconds to make a 10^{-1} diluted solution (10^{-1} tube).

d) Take 0.5 ml of diluted sputum from the 10^{-1} tube, and transfer into the 10^{-2} tube. Vortex for 20 seconds to make a 10^{-2} diluted solution (10^{-2} tube).

e) In the same way as above, dilute serially up to 10^{-5} to make 10^{-3} tube, 10^{-4} tube, and 10^{-5} tube.

③ Inoculate each of serially diluted sputum (10^0, 10^{-1}, 10^{-2}, 10^{-3}, 10^{-4}, and 10^{-5}) onto 4 plates of Selective 7H11 agar with 0.2 ml per plate.

- Long-barrier tips should be used to avoid contact with bacteria.
- To reduce errors as much as possible, change the tip and spreader after a single use.
- If it is difficult to purchase BBL Selective 7H11 agar (Deep Fil), make and use 7H11 (with PACT) agar.

④ Write down sputum ID, Lab No., inoculation date, etc. on a labelling tape. Attach it to a plastic bag.

⑤ After wrapping the inoculated plates with CO_2-permeable tape, put them agar-side down in the plastic bag, and incubate in a 5% CO_2 incubator at 37°C.

⑥ Record sputum inoculation date, contamination check date, and plate reading date in CO_2 Incubator Instrument Inventory Log.

- Contamination check date: 3 days and 7 days after inoculation
- Plate reading date: 21 days ± 3 days after inoculation

2. Colony Counting

① Contamination should be checked 3 days and 7 days after sputum inoculation.

- After 3 days, check mainly for bacterial contamination. After 7 days, check for contamination by spore-forming fungi such as mold.
- Record contaminated plates as "Contam" in Selective 7H11 Agar Colony Counting Results, and mark the contaminated part of colonies with a red oil pen.

② After 14 days, check for additional contamination on Selective 7H11 agar, and mark contaminated parts with a red oil pen. In case of growth of *Mycobacterium tuberculosis*, record as "D (Detected)"in Selective 7H11 Agar Colony Counting Results. Record as ND (Not Detected) in case of no growth.

③ If day 21 is on weekends, count 2-3 days later on days 23 or 24.

④ Check bacterial growth after 21 days, and count colonies before they become too large and merge with adjacent colonies.

a) Record counted numbers of colonies on plates in case of bacterial growth.
b) If the number is more than 200, record as "TNTC (Too numerous to count)".
c) In case of no bacterial growth, record as "ND (Not Detected)".
d) If it is contaminated but colony counting is possible, write down "Contam (n = number of colonies)".

⑤ To save results, take a photo of the plates, and save the file.

a) Place 24 plates at dilutions of 10^0~10^{-5} together so that the labels displaying sputum ID, Lab No., and photo-taking date can be seen, and take a photo and save it.
b) If plates have 20 - 200 colonies, take a photo of each plate separately, and save the file.

3. Report on Final Results

① Select a dilution factor resulting in the number of colonies within the range of 20 - 200 among the plates checked on day 21.

② If there are several dilution plates in the range of 20 - 200 colonies, select a plate with more colonies and mark ( ).

③ Record the calculation processes of 4 count values and calculate CFU/ml values.

●Example: In case that a specimen has been serially diluted with 500 ul, the average of the numbers of colonies grown on a 10^{-5} plate is 20, and the inoculum volume is 0.2 ml each,

20 colonies x (1000 ul/200 ul) x 1/10^{-5} dilution = 1x10^7 CFU/ml

④ Convert CFU/ml values to logarithmic scale, record log CFU/m values, and record the average of 4 log CFU / m values in Average column.

4. Storage of *Mycobacterium tuberculosis*

① For additional experiments such as drug susceptibility testing, select 1-2 plate(s) confirmed with bacterial growth and no contamination, and store in the short-term.

② Record the list in Short-term Storage Log for Mtb Isolates.

5. Reagents and Media Required

① 0.85% saline solution, 1000ml

a) Add 8.5g NaCl to 900 ml D.W, and mix well.
b) Adjust the volume to 1000 ml with D.W.

② Saline-Tween 80, 1000ml

a) Add 2.7g of Tween-80 taking into account its specific gravity, adjust the volume to 1000 ml with 0.85% Saline solution, and mix well.

b) Autoclave and store at 4℃ until use. It can be stored for 6 months from the date of preparation.

③ BBL™ Selective 7H11 agar (Deep Fill), Cat No. 221868

- If it is difficult to purchase BBL™ Selective 7H11 Agar (Deep Fill), prepare 7H11 (with PACT) agar medium as described below, and use it.

④ Preparation of antibiotics PACT

Antibiotics	Stock conc.	Solvent	Working conc.	Comments
Polymixin B (Cat. No. P0145)	200,000 U/ml	Sterile DW	200 U/ml	Prepared at 1 million unit and 1:5 diluted once again
Amphotericin B (Cat. No. A0103)	10 mg/ml	DMSO	10 ug/ml	
Carbenicillin (Cat. No. C0109)	50 mg/ml	Sterile D.W	50 ug/ml	Potency considered (Different depending on Lot No.)
Trimethorprim lactate (Cat. No. T0667)	20 mg/ml	DMSO	20 ug/ml	

a) Purchase products from Sigma Aldrich, and dissolve each antibiotic in an appropriate solvent as described above to prepare antibiotic stock solutions.

b) After filtration through a 0.22 um syringe filter, make 0.5 ml aliquots. Put the aliquots in a sample box labeled with preparation date and self-life, and store at -70°C.

c) The antibiotic stocks can be stored for one year from preparation date.

⑤ Preparation of 7H11(with PACT) medium, 500 ml

a) Put 10.5 g of 7H11 base and 450 ml of D.W. in a 1000 ml bottle, and stir with a magnetic bar.

b) After adding 5 ml of 50% glycerol, mix and autoclave with the magnetic bar inside.

c) After cooling to 55 ℃, add 50 ml of OADC and 0.5 ml each of four antibiotic PACT stock solutions, and stir at room temperature for 1 minute.

- The final concentration of OADC is 10%

d) Pour 20~25 ml each of the medium into a 90 mm petri dish, and wait for 1 hour. After the agar surface dries, label with preparation date and self-life, avoid exposures to light, and store at 4℃.

e) The self-life is 6 months from preparation date.

6. Equipment and Materials Required

① BBL™ Selective 7H11 agar (Deep Fill) or 7H11 (with PACT) agar

② 0.85% Saline-Tween 80 solution

③ Conical tube (15 ml)

④ Vortex mixer

⑤ Pipet aid

⑥ Serological pipette (5 ml)

⑦ $C0_2$ permeable tape

⑧ Plastic bag

⑨ Long, barrier tip (1 ml, 0.2 ml)

⑩ Petri dish (90 mm)

⑪ Spreader

⑫ Stirrer

⑬ Syring filter, Millex LG 0.20 um, PTFE membrane for aqueous or organic solutions

⑭ DMSO

⑮ Sample box for PACT storage

✓ Worksheets to fill out: New Reagents / Media Form
Reagent Preparation Record Form
Deep freezer Temperature Record Form
Incubator Temperature Record Form
$C0_2$ Incubator Instrument Inventory Log
Selective 7H11 agar Colony Counting Results Form
Refrigerator Temperature Record Form
Short-term Storage Log for M.tb isolates

IV. Sputum Storage for Backup and RNA preparation

1. Storage of Sputum for Backup

① Put 1.5 ml of homogenized sputum in a cryovial, and store at -70 °C.

② Record the position of the sample box, sputum ID, Lab No., Cryovial No., storage date, etc. in Sputum Storage Log.

③ Record in Position in Box for Sputum Stored.

2. Pre-treatment before RNA preparation

① Before starting the experiment, set a centrifuge to 4 ℃ in advance.

② Centrifuge homogenized sputum at 3000 g for 15 minutes at 4℃ and discard the supernatant.

③ To wash pellets, add dPBS (pH 7.0) up to 20 ml, and disperse pellets by pipetting. Centrifuge at 3000 g for 15 minutes, 4 ℃, and discard the supernatant.

④ After adding a different volume of Trizol® reagent according to the amount of the remaining pellet, vortex. Transfer 1 ml each to a 2 ml screw cap tube containing 0.1 mm glass beads.

- For example, if the amount of a pellet is less than about 250 ul, add 1 ml of

Trizol. If it is 250 - 500 ul, add 2 ml of Trizol, and if it is 500 - 750 ul, add 3 ml of Trizol.

⑤ Store at 4℃ until delivery to Yonsei University

3. Delivery

① Trizol-treated sputum specimens will be delivered to Yonsei University next time the person in charge of delivery visits.

- Delivery: Room 216, Mirae Hall, Wonju Campus, Yonsei University, 1 Yonseidae-gil, Heungup-myeon, Wonju-si, Gangwon-do
- To: Yeon Kim (Mobile: 010-8907-3224, Office: 033-760-2938)

② Fix Trizol-treated sputum specimens in a styrofoam box containing ice packs, and close the lid.

③ Deliver the triple-packed sputum transport box with a copy of “Certificate of Receipt of Sputum Specimens” and the original of “Certificate of Receipt of Sputum Specimens for mRNA Analysis” to the person in charge of transportation. File a copy of the "Certificate of Receipt of Sputum Specimens for mRNA Analysis" in the Tuberculosis Research Institute.

4. Equipment and Materials Required

① Refrigerated Centrifuge

② Trizol

③ dPBS (pH 7.0)

④ 0.1 mm glass bead (Biospec, No.11079101)

⑤ 2 ml screw cap tube

⑥ Long-barrier tip (l ml)

⑦ Refrigerator

⑧ Cryovial

⑨ Certificate of Receipt of Sputum Specimens for mRNA Analysis

✓ Worksheets to fill out: Refrigerated Centrifuge Temperature Record Form
Reagent Preparation Record Form
Deep Freezer Temperature Record Form
Sputum Storage Log Form
Position in box for Sputum Stored
New Reagents / Media Form

V. Culture Tests in Liquid and Solid Media

1. Decontamination (NaOH)

① Add an equal volume of NALC-NaOH-SC solution to an aliquot of homogenized sputum (5ml) for MGIT/Ogawa culture, vortex for 20 seconds, and leave it to stand for 20 minutes.

② NaOH treatment time should not exceed 20 minutes.

③ Fill sterilized dPBS (pH 7.0) up to 45 ml based on the scale of the 50 ml conical tube, close the lid, and mix by inverting the tube 5-10 times.

④ Centrifuge at 3000 g at 4℃ for 15 minutes, and discard the supernatant in a waste container containing disinfectant.

⑤ Add 1 ml of sterilized dPBS (pH 7.0) and mix well using a 1 ml pipette.

2. Culture Test in Liquid Medium (BACTEC MGIT 960)

① After recording serial numbers of MGIT tubes and sputum information in MGIT Tube Serial Number Log, label the MGI tubes with patient information using a labeling tape.

② Add 15 ml of supplement to a lyophilized MGIT PANTA bottle, and mix well.

③ Add 0.8 ml of the above PANTA-Supplement solution to each labeled MGIT tube.

④ Inoculate 0.5ml of decontaminated sputum into each MGIT tube.

⑤ Close the lid and mix by inverting the tube 5-10 times.

⑥ Scan the bar code of the inoculated MGIT tube into the MGIT 960, place it in a specified position with green light on, and incubate for 42 days.

3. Examination of Growth in Liquid Medium Culture (MGIT tube).

① If MGIT 960 shows a positive signal, take out the tube, check the growth of *Mycobacterium tuberculosis* by visual examination, and record in Log.

② Print the result sheet, sign, and file it.

- If positive response is found within 3-4 days, inoculate 100 ul of the MGIT culture on a LB plate to check whether it is contaminated by other bacteria, and then proceed to the next step.

③ After visual examination and verification, put the MGIT tube back into the MGIT 960 instrument and incubate further for 4-5 days for MPT64 ID.

- The result sheet should be printed out before putting the MGIT tube back.
- If the antigen-antibody reaction test using the MPT64 kit is performed 3 days after examining the growth in MGIT or earlier, the result may be unclear. Therefore, incubate further for 4-5 days after examining the growth in MGIT.

④ After 4-5 days of additional incubation, prepare a MPT64 kit and record sputum ID, Lab No., and date.

⑤ Carefully open the lid of the MGIT tube, take 100 ul of the bacterial culture, and proceed with the MPT64 test.

⑥ Drop the bacteria suspension on a strip, leave it to react for 15 minutes.

⑦ As a control, use negative and positive MGIT tubes inoculated for QC of MGIT tubes. 0

⑧ If the strip shows 2 lines after 15 minutes, it is determined as Mtb-positive whereas if 1 line appears, it is determined as Mtb-negative.

⑨ If result reading is indeterminate due to unclear strip lines, incubate the MGIT tube further, and repeat the MPT64 test.

⑩ Record results in Identification of MGIT Culture Log and take a picture for storage.

4. Culture Test on Solid Medium (Ogawa)

① Before inoculation, remove condensed water in the tube. Let the condensed water flow down along the slope of the medium by bringing the tube with the

slope side down into contact with sterile gauze.

② Record sputum ID, Lab No., and date on the lid of Ogawa medium.

③ Inoculate evenly by spreading 0.1 ml of decontaminated sputum using a barrier tip.

④ Keep the lid slightly opened and incubate in a tilted position at 37°C for 1-2 day(s) so that the inoculum can be fully absorbed.

⑤ Check for complete absorption of inoculum and contamination 3-5 days after inoculation. For the well absorbed culture bottle, close the lid and incubate.

⑥ Read once a week from week 2 thereafter, and incubate up to 8 weeks to verify negative reading.

⑦ Record culture results in Ogawa Culture Log.

5. Short-term Storage of Culture Medium after Culture Test

① Among the culture tubes that have been tested for liquid and solid medium culture, positive cultures are stored at 4°C for one month.

② Record the list in Short-term Storage Log for Mtb Isolates.

6. Reagents Required

① 2.9% Sodium citrate, 500ml
- Dissolve 14.5 g of sodium citrate in D.W, and adjust the volume to 500 ml. After autoclaving, divide into 25 ml each, and store at 4°C.

② 6% NaOH, 500ml
- Dissolve 30 g of NaOH in D.W, and adjust the volume to 500 ml. After autoclaving, divide into 25 ml each, and store at 4°C.

③ NALC-NaOH-SC, 50ml
- Immediately before use, mix an equal volume (25 ml each) of 6% NaOH and 2.9% sodium citrate solutions. To this NaOH-sodium citrate solution, mix NALC to 0.5% (0.25 g).

7. Equipment and Materials Required

① BACTEC MGIT 960 Machine
② MGIT Growth Indicator Tube
③ MGIT Growth Supplement
④ MGIT PANTA
⑤ Ogawa Medium
⑥ Refrigerated Centrifuge
⑦ Micropipetors (200 ul and 1000 ul)
⑧ Micropipette Tip (20-200 ul and 200-1000 ul)
⑨ SD TB MPT64 Rapid Kit

✓ Worksheets to fill out: Reagent Preparation Record Form
New Reagents/Media Form
MGIT 960 Machine Maintenance Form
Incubator Temperature Record Form
Refrigerated Centrifuge Temperature Record Form
MGIT tube Medium QC Form
Ogawa/L-J Medium QC Form
Ogawa Culture Log Form
MGIT Tube Serial Number Log Form
Identification of MGIT 960 Culture Log Form
Short-term Storage Lo9 for Mtb Isolates Form

VI. Subculture and Long-term Storage of Strains

Purpose

Specimens of visit number 2 and 9 (EBA 0 and 14) need to be stored in the long-term since a drug susceptibility test is required to perform. *Mycobacterium tuberculosis* grown in solid and liquid medium is subcultured in L-J medium and stored at -70°C in the long-term.

1. Subculture of Strains

① For Visit number 2 and 9 (EBA 0 and 14), drug susceptibility testing should be performed. Therefore, the strains obtained from solid and liquid culture tests are subcultured.

- The strains from Visit number 2 and 9 are tested for drug susceptibility on the same day to compare resistance Therefore, if possible, inoculate on the same day to match the two strains to similar state.

② Record sputum ID, Lab, and date on 2 tubes of L-J solid medium.

③ Subculture the positive strains of tuberculosis bacteria grown in solid medium (Selective 7H11 agar, Ogawa) or liquid medium (MGIT tube).

- Using a disposable loop, pick as many colonies as possible from the solid medium with tuberculosis bacteria, and spread evenly on the slope of the L-J medium.
- Using a long-barrier-tip, take 0.1 ml of bacterial culture at the bottom of the MGIT tube and inoculate evenly by spreading on the slope of the medium.

④ Keep the lid slightly opened and incubate in a tilted position at 37°C for 1-2 day(s) so that the inoculum can be fully absorbed.

⑤ Check complete absorption of inoculum and absence of contamination 3 days after inoculation. For the well absorbed culture bottle, close the lid and incubate for 3-4 weeks.

⑥ Record the information on inoculated strains in Subculture Log for Mtb Isolates

2. Storage of Strains

① After 3-4 weeks, take as many colonies as possible of tuberculosis bacteria grown on the medium using a disposable loop, and make one stock in each of 10% skim milk and 10% glycerol.

② Tightly close the lids of cryovials containing tuberculosis bacteria, and disperse large aggregates by vortexing for 30 seconds.

③ Record in Long-term Freezer Storage Log for Mtb Isolates and Position in Box for Mtb Isolates Log.

3. Reagents Required

① 10% glycerol, 100 ml

a) Add 12.75 g of glycerol taking into account the specific gravity of glycerol, adjust the volume to 100 ml with D.W, and mix well.

b) Aliquot 1.0 ml each into cryovials, autoclave, and store in a refrigerator.

c) Randomly select 2 tubes out of the aliquots, and spread 0.1 ml of 10% glycerol on LB medium to check for contamination. If any contamination is found, discard the aliquots and prepare again.

d) The self-life is 6 months.

② 10% skim milk, 100 ml

a) Weigh 10 g of skim milk powder on a scale, and completely dissolve in 90 ml of D.W.

b) Add D.W. up to 100ml, and aliquot 1.0 ml each into cryovials.

c) Autoclave and store in a refrigerator.

d) Select randomly 2 tubes out of the aliquots, and spread 0.1 ml of 10% skim milk on LB plates to check on contamination. If any contamination is found, discard the aliquots and prepare again.

e) The self-life is 6 months.

4. Equipment and Materials Required

① 1.5 ml Cryovial

② Sample box

③ 10 ml Serological disposable pipette, aid

④ Disposable loop

⑤ L-J medium

⑥ LB plate

✓ Worksheets to fill out: Long-term Freezer Storage Log for M.tb Isolates Form

Subculture Log for M.tb Isolates Form

Position in Box for M.tb Isolates Form

10% glycerol/Skim milk QC Form

New Reagents/Media Form

Reagent Preparation Record Form

VII. First and Second Anti-tuberculosis Drug Susceptibility (M-kit)

1. Specimens

① This test is performed with *Mycobacterium tuberculosis* cultures within 15 days after growth has been positively confirmed on solid medium.

② It is recommended to use primary culture. However, subcultures afterwards can be used.

③ In case of insufficient bacterial growth (<10 colonies), drug susceptibility testing should not be continued.

2. Preparation of Bacterial Suspension

① Add 50 ul of dPBS (pH 7.0) to the round-bottle tube containing glass beads.

② Take bacteria from the slope of the medium with a loop and carefully disperse the bacteria on the glass beads.

③ Close the cap and vortex with a vortex mixer until the bacteria attached to the

tube are not seen.

④ Leave it to stand at room temperature for 10 minutes.

3. Dilution of Bacterial Suspensions

① Adjust the concentration of bacteria suspension to McFarland No. 1.0 by adding dPBS (pH 7,0).

② Leave it to stand for 20 minutes.

③ Put 4.5 ml of dPBS (pH 7,0) to a 15 ml conical tube, and add 0.5 ml of the above bacteria suspension to make a 1:10 dilution.

4. Inoculation and Cultivation

① Leave the M-kit at room temperature before use, and remove the condensed water in advance.

② After vortexing the bacteria suspension, inoculate 25 ul of it to the medium containing drugs in the M-kit using a step-syringe (1.5 ml).

③ Stir the M-kit so that bacterial cells spread evenly, and then wrap the sides with paper tape.

④ The inoculated M-kit is incubated at 37℃ and observed every week.

⑤ Either sensitivity (S) or resistance (R) is determined after incubation for 4 weeks. However, incubation time can be prolonged up to 6 weeks if bacterial growth is not sufficient.

5. Placement and concentrations (ug/mL) of drugs in the M-Kit susceptibility medium

Control		INH 0.2	RFP 40	SM 4	EMB 2	
Control	INH 1	INH 0.2	RFP 40	SM 10	EMB 2	
KM 30	CPM 40	PTH 40	CS 30	PAS 1	OFX 4	
MOX 2	AMK 30	LEV 2	RBT 20	PNB 500	LIN 1	

① Description of antibiotic abbreviations

INH: Isoniazid ; RFP: Rifampicin ; SM: Streptomycin ; EMB:. Ethambutol ; KM: Kanamycin ; CPM: Capreomycin ; PTH: Prothionamide ; CS: Cycloserine ; PAS: Paraaminosalicylic acid ; OFX: Ofloxacin ; MOX: Moxifloxacin ; AMK: Amikacin ; LEV: Levofloxacin ; RBT: Rifabutin ; PNB: Pafa-nitribenzoic acid ; LIN: Linezolid

② Critical concentration

INH 0.2 ug/ml, SM 10 ug/ml

③ Stored at 4 ℃. The self-life is 6 months from preparation date.

6. Control Samples

① When conducting a drug susceptibility test using a M-kit, always perform *M. tuberculosis* strain H37Rv ATCC 27294 as a control.

② The number of passaging of H37Rv stock (Passage P0) does not exceed 5.

③ Preparation and inoculation of bacteria suspension proceed in the same manner as for samples.

④ The results of the drug susceptibility test in M-kit should be “susceptible” for all drugs.

7. Equipment and Materials Required

① M-kit

② 2mm Glass bead
③ Round bottle, screw cap tube
④ Loop
⑤ Vortex mixer
⑥ Conical Tube(15ml)
⑦ Paper Tape
⑧ Pipet aid
⑨ Serological pipette (1ml and 5ml)
⑩ Step-syringe, 1.5 ml tip
⑪ McFarland Standard No. 1.0
⑫ dPBS (pH7.0)

✓ Worksheets to fill out: Incubator Temperature Record Form
DST Log Using M-kit
M-kit DST QC Form

VIII.Minimal Inhibitory Concentration (MIC) Test of LCB01-0371

1. Drug LCB01-0371 Placement by Concentrations (ug/ml)

Control(H37Rv)	0.25	0.5	1.0	2.0	4.0	8.0
Control(dPBS)						
Control(Visit No. 2)	0.25	0.5	1.0	2.0	4.0	8.0
Control(Visit No. 9)	0.25	0.5	1.0	2.0	4.0	8.0

① Prepare L-J media containing LCB01-0371 at 0.25, 0.5, 1.0, 2.0, 4.0, and 8.0 ug/ml.

- These media can be stored at 4℃ and used for 6 months.

② Inoculate bacteria suspensions of Visit No. 2 and 9 (EBA 0 and 14) in parallel in a single panel.

③ Use the strain H37Rv for internal controls. For negative controls, inoculate dPBS (pH 7.0) used for dilution of bacteria suspensions.

2. Culture and Inoculation

① Use cultures within 15 days after confirmation of bacterial growth.

② After preparing bacteria suspension at McFarland No. 1.0, dilute in dPBS (pH 7.0) at 1:10, and leave it to stand for 10 minutes.

③ Remove the condensed water from the medium containing the drug LCB01-0371.

④ Inoculate 25 ul of the diluted suspension in L-J medium and incubate at 37 ℃.

⑤ Examine the inoculated medium every week and read after incubation for 4 weeks (some strains grow very slowly and require additional 2 weeks for cultivation. Therefore, make the first treading after 4 weeks, report the results, and make the final reading on week 6.

3. Experiments with Control Strains

① Whenever conducting a susceptibility test with the drug LCB01-0371, perform the test of *M. tuberculosis* strain H37Rv ATCC27294 in parallel as a control.

② Proceed to preparation of bacteria suspension and inoculation in the same way as for specimens.

4. Equipment and Materials Required

① L-J medium (with LCB0I-0371 at 0.25, 0.5, 1.0, 2.0, 4.0, and 8.0 ug/ml)

② 2 mm Glass beads

③ Round-bottle screw cap tube

④ Loop

⑤ Pipet aid

⑥ Serological pipette (5 ml)

⑦ Micropipetors (200 ul)

⑧ Micropipette tips (20-200 ul)

⑨ Step-syringe, pipette

✓ Worksheets to fill out: Incubator Temperature Record Form

MIC test Log & QC using L-J medium(LCB01-0371) Form

LCBOI-0371 Inventory L09 Form

IX. Quality Control (QC)

1. Practice of Quantitative Culture

Before the initiation of a full-scale clinical study, perform the experiment below by diluting tuberculosis bacteria in various ways and see whether the result CFU/ml values are consistent. To increase experimental accuracy and experimenters' proficiency, repeat 3 times and perform once a month after the initiation of a full-scale clinical trial.

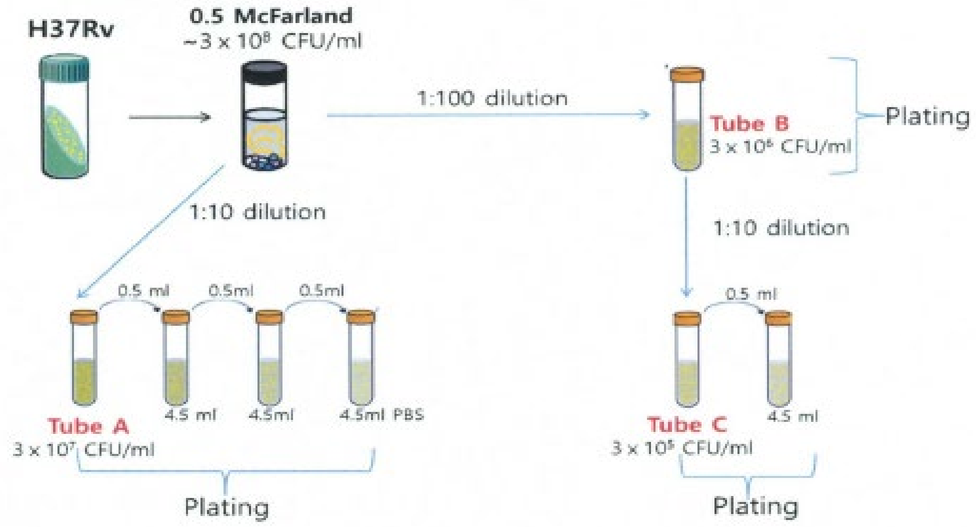

2. Practice of Quantitative Culture (Before Initiation of Clinical Study)

① Preparation of Bacterial Suspensions

a) Subculture H37Rv (ATCC 27294) in L-J medium.

b) Incubate at 37℃ for 20~30 days.

c) Take bacteria from the slope of the medium with a loop and disperse the bacteria on glass beads.

- Disperse aggregated tuberculosis bacteria into single cells as completely as possible to obtain consistent results.
- Excessive vortexing can affect viability of tuberculosis bacteria.

d) Prepare bacterial suspension at McFarland No. 0.5, aliquot 1.5 ml each, and store at -70℃ (The self-life is 6 months).

- McFarland No. 1 based on *E. coli* has been reported to be 3.0 x 10^8 CFU/ml. However, this number is changed if based on tuberculosis bacteria rather than *E. coli*.
- Whereas the size of *E. coli* is 1-2 um, the size of *Mycobacterium tuberculosis* is 3.5-4 um. *Mycobacterium tuberculosis* is not only more than twice larger than *E. coli*, but also can be even larger in actual size because it does not exist as single cells due to fatty acid in the cell wall.
- According to an article that compared OD values to turbidities determined by McFarland No. in *Mycobacterium tuberculosis*, McFarland No. 1 based on *Mycobacterium tuberculosis* is reported to be 1.97 x 10^6 CFU/ml.
- Therefore, experiments are performed based on actual numbers of bacteria after preparing a bacteria suspension of *Mycobacterium tuberculosis* at McFarland No. 0.5 and determining the number of bacteria in it..
- For example, if a bacteria suspension prepared at McFarland No. 0.5 is measured to be 1.0x 10^8 CFU/ml, bacteria numbers in Tube A~C should be calculated based on this actual number.

e) Add 0.5 ml of bacteria suspension at McFarland No. 0.5 to 4.5 ml of dPBS (pH 7.0) (1:10 dilution), vortex for 20 seconds, and leave it to stand for 10 minutes (<u>Tube A: 10^7 CFU/ml of MTB</u>).

f) Vortex again the bacteria suspension at McFarland No. 0.5 and add 0.1 ml to 9.9 ml of dPBS (pH 7.0). After vortexing for 20 seconds, leave it to stand for 10 minutes (1:100 dilution) (<u>Tube B: 10^6 CFU/ml of MTB</u>).

g) Add 0.5 ml of the above 1:100 diluted bacteria suspension (Tube B) to 4.5 ml of dPBS (pH 7.0), vortex for 20 seconds, and leave it to stand for 10 minutes (1:1,000 dilution) (<u>Tube C: 10^5 CFU/ml of MTB</u>).

h) Dilute 0.5 ml of Tube A bacteria suspension to 4.5 ml of dPBS (pH 7.0) (Tube A / 10^{-1}). Dilute Tube A / 10^{-1} bacteria suspension once more in 4.5 ml of dPBS (pH 7.0) (Tube A / 10^{-2}). Repeat this procedure to make Tube A / 10^{-3} bacteria suspension.

i) Dilute 0.5 ml of Tube C bacteria suspension in 4.5 ml of dPBS (pH 7.0) (Tube C/ 10^{-1}).

② Inoculation and Cultivation

a) Prepare 7H11 agar medium for all cell densities 2 plates each and label all.

b) Inoculate all diluted bacteria suspensions 0.2 ml each using a spreader.

c) After sealing the inoculated media with $C0_2$-permeable tape, put in a plastic bag, and incubate in inverted positions at 37℃.

③ Result Calculation and Acceptance Range

a) Select a dilution factor displaying the number of colonies in the range of 20-200 among the plates inoculated after dilution by Tube A, B, and C methods. If there are more than 2 different dilution factors in this range, select one with more colonies.

b) Count colonies on the plates inoculated according to Tube A, B, and C methods, and calculate the average of the 3 values.

c) Calculate CFU/ml values.

- Example: If the average of the numbers of colonies grown on the 10^{-5} plates after serial dilution is 20 and the inoculum volume is 0.2 ml each,
  20 colonies x (1000 ul/200 ul) x $1/10^{-5}$ dilution = $1x10^{7}$ CFU/ml

d) Compare CFU/ml values for each concentration.

e) When CFU/ml values from Tube A/10^{-1} and Tube B, Tube A/10^{-2} and Tube C, or Tube A/10^{-3} and Tube C/10^{-1} are compared, the two CFU/ml values of each pair should be theoretically the same.

f) When comparing the CFU/ml value obtained by Tube B or Tube C method to that by Tube A method, the difference should be within 20%, which is an acceptance range.

- If the difference does not meet the acceptance range, repeat the experiment once.
- In cases that the result of the second experiment does not meet the acceptance range, check the concentration of the stock and accuracy of the initial inoculum, etc.

④ Practice of Quantitative Culture (After Initiation of Clinical Study)

a) Using the strain prepared for practice of quantitative culture, perform the same experimental procedure as above. Check whether the CFU/ml values obtained are within the acceptance range.

- Compare CFU/ml values from Tube A/10^{-1} and Tube B, Tube A/10^{-2} and Tube C, and Tube A/10^{-3} and Tube C/10^{-1}. When comparing the CFU/ml value obtained by Tube B or Tube C method to that by Tube A method, the difference should be within 20%, which is an acceptance range.
- If the difference does not meet the acceptance range, repeat the experiment once.
- In cases that the result of the second experiment does not meet the acceptance range, check the cell density of the stock and accuracy of the initial inoculum, etc.

✓ Worksheets to fill out: <u>Deep freezer Temperature Record Form</u>
<u>Incubator Temperature Record Form</u>
<u>Exercise culture Colony Counting Results Form</u>
<u>Mtb(H37Rv) Stock Log Form</u>

3. **MGIT Time To Detection (TTD) Standardization**

Before the initiation of a full-scale clinical study, check whether inoculated tuberculosis bacteria exhibit consistent TTD values by dilution factors when diluted as below and inoculated into MGIT tubes. Repeat 4 times before the initiation of a clinical trial to determine the acceptance range of TTD and perform once a month after the initiation of a clinical trial.

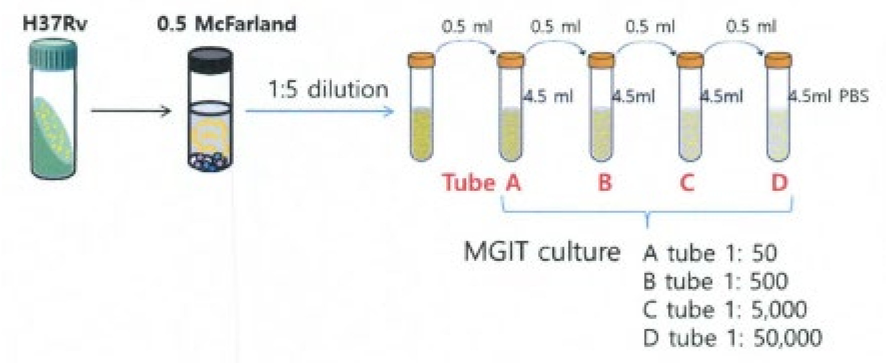

① Preparation of bacterial suspensions

a) Subculture H37Rv (ATCC 27294) in L-J/Ogawa medium or 7H9 medium.

b) Take bacteria from the slope of the medium with a loop and disperse the bacteria on glass beads.

c) Prepare bacterial suspension at McFarland No. 0.5, aliquot 1.5 ml each, and store at -70℃ (The self-life is 6 months).

d) Add 1 ml of bacteria suspension at McFarland No. 0.5 to 4 ml of dPBS (pH 7.0) (1:5 dilution), vortex for 20 seconds, and leave it to stand for 10 minutes (1:5 dilution).

e) Add 0.5 ml of the above 1:5 diluted bacteria suspension to 4.5 ml of dPBS (pH 7.0), vortex for 20 seconds, and leave it to stand for 10 minutes (A tube 1:50 dilution).

f) Add 0.5 ml of the above 1:50 diluted bacteria suspension to 4.5 ml of dPBS (pH 7.0), vortex for 20 seconds, and leave it to stand for 10 minutes (B tube 1:500 dilution).

g) Repeat the above procedure to 1:50,000 dilution (C tube 1:5,000, D tube 1:50,000).

② Inoculation

a) Add 0.8 ml of OADC to each MGIT tube.

b) Add 0.5 ml each of the diluted bacteria suspensions A~D tubes, close the cap, and mix by inverting tubes several times.

c) Scan the inoculated MGIT tubes into MGIT 960, place in designated positions with green light on, and incubate.

③ TTD standardization

a) Repeat the above procedure 4 times within 7 days.

b) For TTD values obtained from 4 repeated experiments, calculate and record the acceptance range (20%) of the average values by A~D tubes.

- Example:

A tube 1 : 50 → 4: 14 ~ 6 : 21

B tube 1 : 500 → 6; 10 ~ 9 : 15

C tube 1 : 5,000 → 10; 9 ~ 15 : 14

D tube 1 : 50,000 14 : 11 ~ 21 : 16

④ Monthly MGIT TTD (after Initiation of Clinical trial)

a) Use the MTB stock strain prepared for practice of quantitative culture.
b) Dilute the bacteria suspension as above in TTD standardization procedure and inoculate MGIT tubes.
c) Check whether TTD values obtained from each dilution are within 20% of the average of TTD standardization setting.
d) If the result is not within the acceptance range, repeat the experiment once.
e) In cases that the result of the second experiment is not within the acceptance range, check the cell density of the stock and accuracy of the initial inoculum.

✓ Worksheets to fill out: MGIT 960 machine maintenance Form

MGIT TTD Standardization Form

Mtb(H37Rv) Stock Log Form

4. BBL™ Selective 7H11 agar (Deep Fill)

BBL™ Selective 7H11 agar (Deep Fill) or prepared 7H11 (with PACT) medium should be used after sterility testing and MTB growth testing. These tests aim to evaluate potencies of antibiotics PATC and check whether the prepared 7H11 medium is appropriate for the growth of *Mycobacterium tuberculosis* using *E. coli*.

① Selectivity test

a) Test 1~3% of purchased or prepared media to confirm efficacies of antibiotics that suppress the growth of contaminants.
b) Prepare *E. coli* grown in LB broth adjusted to McFarland No. 0.5, aliquot 0.1 ml each into cryovials, and store at -70℃ (These stocks can be stored for 12 months).
c) To evaluate the efficacy of Selective 7H11 agar, take out an *E. coli* stock, inoculate in LB broth, and incubate overnight at 37°C.
d) Dilute overnight cultured *E. coli* in dPBS (pH 7.0) to a cell density of 1×10^4 CFU/ml.
e) Inoculate 200 ul each onto LB plates and Selective 7H11 agar, tape the plates, put in a plastic bag, and incubate in inverted positions at 37℃.
f) After the bacteria grow, count colonies and calculate inhibition rates as follows.
    - [Number of colonies grown on Selective 7H11 agar] / [Number of colonies grown on LB plate]
g) Acceptance range
    - The inhibition rate should be 70% or more.
    - If less than 70%, re-test with new plates. If still less than 70%, discard the medium.

② Sterility test and MTB growth test

a) Use 1-3% of newly purchased media.

b) Cultivate MTB in the newly purchased medium and the previously used lot of medium and incubate plates without inoculation for the sterility test.

c) Use the strain prepared for practice of quantitative culture. Serially dilute to 10^{-4} and 10^{-5} (the range of serial dilution producing an appropriate number of colonies for counting), and inoculate with each diluted culture. After incubation, count colonies and calculate averages.

d) Check the growth rate and colony size of MTB on the newly purchased medium.

e) Compare the colony number, cell size, and growth rate on the newly purchased medium to those on the previously used medium and record the result in Selective 7H11 agar QC Form.

③ Acceptance range

a) The result values should be within 20% (acceptance range).

b) If the result values are not within the acceptance range, re-test. If the result is repeated, discard the medium.

✓ Worksheets to fill out: Selective 7H11 agar QC Form
Mtb (H37Rv) Stock Log Form
E coil Stock Log Form
Deep freezer Temperature Record Form

5. Culture Test in Liquid Medium (MGIT Growth Tube)

① Perform this test every time a new batch of MGIT growth tubes is received.

② For a positive control, prepare a bacteria suspension of H37RV *Mycobacterium tuberculosis* at a turbidity of McFarland No. 0.5. After 1:500 dilution, inoculate 0.5ml.

③ For negative control, inoculate 0.5 ml of dPBS (pH 7.0).

④ After cultivation, print the result paper and file it. Check whether the TTD value is kept consistent.

⑤ For the acceptance range of TTD values, refer to TTD standardization values.

✓ Worksheets to fill out: MGIT 960 machine maintenance Form
MGIT tube medium QC Form
Mtb(H37Rv) Stock Log Form

6. Solid Medium (Ogawa, L-J) Culture Test

① Perform this test every time a new batch of solid media is received.

- Alternatively, receive and use the 'Medium Quality Control Register' conducted by the Department of Diagnostic Tests and Medicine.

② Adjust the turbidity of H37RV tuberculosis bacteria to McFarland No. 0.5. After 1:100 dilution, inoculate 0.1 ml and incubate.

③ For a negative control, add 0.1 ml of either dPBS (pH 7.0) or 7H9 broth.

④ After incubation for 6 to 8 weeks, check whether positive controls show confluent

bacterial growth.

- ✓ Worksheets to fill out: Incubator Temperature Record Form
  Ogawa/L-J medium QC Form
  Mtb(H37Rv) Stock Log Form

X. Xpert Mtb/RIF Test

1. Preparation of Equipment

① After turning on the GeneXpert instrument, turn on the notebook.

② Click Cepheid to automatically launch the GeneXpert DX software.

③ "Do you want to perform Database Management task?" and other windows open. Then, click the No button.

④ When the device and computer are normally connected, self-testing proceeds. When self-testing of the instrument is completed, check whether the Module Progress has changed from Booting to Available.

2. Preparation of Specimens (Sputum)

① Add Sample Reagent (SR) twice the amount of sputum in a sample container.

② After vortexing for 10 seconds, leave it at room temperature for 15 minutes. At this step, vortex once every 5 minutes.

③ After ensuring that the sample is completely liquefied, take 2 ml and put in the cartridge.

3. Preparation of Cartridges

① Take out the cartridge and reagent from the packaging container.

② Using a provided pipette, carefully aspirate the sample to the mark on the pipette.

③ Open the lid of the cartridge, put it in the chamber, and close the lid.

4. Preparation to Start the Test

① Click “Create Test” in the GeneXpert System Window.

② Enter Patient ID by scanning or manually (optional).

③ Enter Sample ID by scanning or manually (optional).

④ When the "Scan Cartridge Barcode dialog" window appears, scan the barcode on the cartridge. Using the barcode information, the software automatically enters Lot ID of the reagents, cartridge serial number, and expiration date.

⑤ Click the Start Test button.

⑥ Open the selected module door and insert the cartridge.

⑦ When a green light is blinking, close the module door. Then the test starts. When the test is completed, the green light turns off and the door opens automatically.

5. Organization of Equipment

① Click the X button to close the GeneXpert DX software, and click the No button when "Do you want to perform Database Management task?" and other windows appear.

② After shutting down the notebook, turn off the device.

6. Expected Results and Reporting

① Target: rpoB

② Effective range of Probe Ct

Probe	Probe A	Probe B	Probe C	Probe D	Probe E	SPC
Effective Range	3~39	3~39	3~39	3~36	3~36	3~38

③ Criteria for TB-positive response

a) In cases that Ct values of 2 or more out of 5 probes are within the effective range

- Example)

Probe	A	B	C	D	E	SPC
Ct	22.7	0	21.9	37.1	22.0	0

: Ct values of Probe A, C, and E (more than 2) are in the effective range: MTB detected

b) In cases that the smallest difference of Ct values between probes is less than 2

- Example)

Probe	A	B	C	D	E	SPC
Ct	22.7	22.3	21.9	21.6	22.0	29.7

: 21.9 (Ct of Probe C) - 21.6 (Ct of Probe D) = 0.3 < 2: MTB detected

c) The Ct values are classified as high, medium, low, and very low. The criteria based on Ct values are as follows.

MTB result	Ct range
High	<16
Medium	16~22
Low	22~28
Very LoW	>28

④ Criteria for RIF

a) The largest of Ct values - the smallest of Ct values ≤ 4: Rifampin-sensitive

b) The largest of Ct values - the smallest of Ct values > 4: Rifampin-resistant

7. Determination of Results

① MTB detected; RIF resistance detected

a) Ct values of 2 or more out of 5 probes are within the effective range.

b) The smallest difference of Ct values between probes is less than 2.

c) Difference between the largest and smallest Ct values of probes is more than 4.

- Example)

Probe	A	B	C	D	E	SPC

Ct	26.7	30.5	29.9	31.1	29.8	25.7

: 31.1 (the largest Ct value) - 26.7 (the smallest Ct value)

= 4.4 > 4 : RIF resistance detected

d) SPC: SPC is not applied as SCP amplification competes with target amplification.

② MTB detected ; RIF resistance not detected

a) Ct values of 2 or more out of 5 probes are within the effective range.

b) The smallest difference of Ct values between probes is less than 2.

c) Difference between the largest and smallest Ct values of probes is less than 4.

- Example)

Probe	A	B	C	D	E	SPC
Ct	23.9	23.6	23.2	22.2	22.8	0

: 23.9 (the largest Ct value) - 22.2 (the smallest Ct value)

= 1.7 < 4 : RIF resistance not detected

d) SPC : SPC is not applied as SCP competes with target during amplification.

③ MTB detected ; RIF Indeterminate

a) Ct values of 2 or more out of 5 probes are within the effective range.

b) The smallest difference of Ct values between probes is less than 2.

c) The smallest Ct value > [the largest effective Ct value of a probe out of the effective range - 4]

- Example 1)

Probe	A	B	C	D	E	SPC
Ct	37.7	35.2	34.4	35.5	36.9	29.7

: 34.4(the smallest Ct value) > 32.0 [36.0 (the largest effective Ct value of Probe E out of the effective range) - 4]

④ MTB not detected

a) Ct values of 5 probes are ineffective, or only one Ct value is within the effective range.

b) SPC should be amplified, and its Ct value should be within the effective range.

- Example)

Probe	A	B	C	D	E	SPC
Ct	0	0	0	0	0	29.7

: Ct values of 5 probes are out of the effective range, and the Ct value of SPC is within the effective range

: MTB not detected

⑤ INVALID

a) The existence of a target cannot be verified because Ct values of a target and SPC are out of the effective range.

b) Cause: - Test not properly conducted.

- PCR inhibited.

c) Proceed retest according to the re-test procedure.

⑥ ERROR

a) The existence of a target cannot be verified because the test fails to proceed due to “Probe check fail” or a problem with system components.

b) Proceed according to the re-test procedure.

⑦ NO RESULT

a) The test result is insufficient (In cases that the test is arbitrarily stopped during the test).

b) The existence of the target cannot be verified because the test has not been proceeded to the end.

c) Proceed according to the re-test procedure.

8. Description of Retest Situation

① INVALID: In cases that Ct values of the target and SCP are not within the effective range, the test is not conducted properly, or PCR is inhibited.

② ERROR: In cases of Probe Check Fail, Pressure limit exceeded, or abnormal position of valve, it occurs, and the test is stopped.

③ NO RESULT: In cases that the test result is insufficient (Example: When the test is arbitrarily stopped during the test).

9. Retest Procedure

① Collect a new specimen from the patient.

② If a new specimen cannot be collected, use the remaining specimen.

a) Less than 5 hours after Sample Reagent treatment

- Take 2 ml of the Sample Reagent-treated specimen and put into "S” Chamber of a new cartridge.

b) More than 5 hours after Sample Reagent treatment

- Mix fresh Sample Reagent and Sputum (Sample Reagent-untreated) at the ratio of 2:1.
- Incubate at room temperature for 15 minutes, and ensure complete liquefication.
- Take just 2 ml and put into "S” Chamber of a new cartridge.

10. Equipment and Materials Required

① GeneXpert device

② Xpert MTB/RIF cartridge

③ Vortex mixer

④ Conical tube (50 ml)

⑤ Pipet aid

⑥ Serological pipette (5 ml)

✓ Worksheets to fill out: Xpert Results Log Form

XI. INH Susceptibility Test by Line Probe Assay

1. **DNA Extraction Using GenoLyse® (HAIN Lifescience)**

① When using specimens from patients, transfer 500 ul of a decontaminated specimen to a labeled 2,0 ml screw cap tube.

② Centrifuge at 10,000 g for 15 minutes using an aerosol-tight rotor centrifuge.

③ After discarding the supernatant disperse the pellet in 100 ul of Lysis Buffer [A-LYS] by vortexing.

④ Incubate for 5 minutes in a water bath set at 95℃.

⑤ Add 100 ul of Neutralization Buffer [A-NB] and vortex for 5 seconds.

⑥ After centrifugation at the full speed for 5 minutes, separate the supernatant. The supernatant is set aside for PCR.

2. **DNA Amplification Using Qiagen HotStarTaq DNA polymerase**

① Make Amplification Mix and dispense into each tube.

a) 35 ul PNM

b) 5 ul 10XPCR Buffer for HotStarTaq

c) 2 ul 25 mM $MgCl_2$ Solution

d) 0.2 ul (1 U) HotStarTaq

e) 3 ul D.W

② Dispense 5 ul of deionized water into the negative control tube and 5ul of the extracted DNA into the test tubes.

③ Amplification

95^0C 15 min	1 Cycle
95^0C 30 sec	10 Cycles
58^0C 2 min	
95 ^{0}C 25 sec	30 cycles
53^0C 40 sec	
70^0C 40 sec	
70^0C 8 min	1 Cycle

3. **Reverse Hybridization**

① Put 20 ul of Denaturation Solution (DEN) into the wells to be used.

② Add 20 ul of amplification products into each well, mix well, and leave it to react for 5 minutes.

③ Add 1 ml of pre-warmed Hybridization Buffer (HYb), and gently shake the tray until the solution is uniform in color.

④ Dip the strip in each well.

⑤ Put the tray in a shaking water bath set at 45° C and leave it to react for 30 minutes.

⑥ Discard the Hybridization Buffer

⑦ Add 1 ml of Stringent Wash Solution (STR) to each strip and leave it to react in

a 45° C. shaking water bath for 15 minutes.

⑧ Discard the Stringent Wash Solution.

⑨ Add 1 ml of Rinse Solution (RIN), leave it to react in a shaking water bath for 1 minute, and discard the solution.

⑩ Add 1 ml of diluted CON-C solution (CON-C 10ul in CON-D I ml) to each strip, leave it to reacted for 30 minutes in a shaking water bath.

⑪ Discard the solution, wash for about 1 minute with 1 ml of Rinse Solution (RIN), and wash once more with 1 ml of deionized water (use a shaking water bath).

⑫ Add 1 ml of diluted SUB-C solution (10 ul of SUB-C in 1 ml of SUB-D) to each strip. Avoid exposures to light and leave it to react in a shaking water bath.

⑬ When bands appear clear, stop the reaction and wash with deionized water.

⑭ Take out the strip from the tray with forceps, put it between two sheets of absorbent paper, and dry completely.

⑮ Using an evaluation sheet, place the strip in line with CC and AC bands, and analyze results. Attach the strip to the evaluation sheet and store it.

4. Result Analysis (In cases that INH resistance has been confirmed in MTBDRplus Assay)

① A case of katG mutation is regarded as resistant, and the patient is excluded.

② In the case of inhA mutation, proceed with the direction of keeping the patient registered.

③ If the katG mutation band or the InhA mutation band is not clear, collect high-quality sputum and repeat the test once more the next day.

5. Equipment and Materials Required

① GenoType MTBDRplus

② GenoLyse®

③ Qiagen HotstarTaq DNA Polymerase

④ Shaking Water Bath

⑤ PCR machine

⑥ Centrifuge

⑦ Screw Cap Tube

⑧ Eppendorf Tube

⑨ Micropipetors

⑩ Micropipette tips (200 ul and 1000 ul)
